# Association between physicians’ interaction with pharmaceutical companies and their clinical practices: A systematic review and meta-analysis

**DOI:** 10.1371/journal.pone.0175493

**Published:** 2017-04-13

**Authors:** Hneine Brax, Racha Fadlallah, Lina Al-Khaled, Lara A. Kahale, Hala Nas, Fadi El-Jardali, Elie A. Akl

**Affiliations:** 1 Faculty of Medicine, Université Saint Joseph, Beirut, Lebanon; 2 Center for Systematic Reviews of Health Policy and Systems Research (SPARK), American University of Beirut, Beirut, Lebanon; 3 Department of Pediatrics and Adolescent Medicine, Faculty of Medicine, American University of Beirut, Beirut, Lebanon; 4 Department of Internal Medicine, American University of Beirut, Beirut, Lebanon; 5 Faculty of Medicine, University of Damascus, Damascus, Syria; 6 Department of Health Management and Policy, American University of Beirut, Beirut, Lebanon; 7 Department of Clinical Epidemiology and Biostatistics, McMaster University, Hamilton, ON, Canada; University of British Columbia, CANADA

## Abstract

**Background:**

Pharmaceutical company representatives likely influence the prescribing habits and professional behaviors of physicians. The objective of this study was to systematically review the association between physicians’ interactions with pharmaceutical companies and their clinical practices.

**Methods:**

We used the standard systematic review methodology. Observational and experimental study designs examining any type of targeted interaction between practicing physicians and pharmaceutical companies were eligible. The search strategy included a search of MEDLINE and EMBASE databases up to July 2016. Two reviewers selected studies, abstracted data, and assessed risk of bias in duplicate and independently. We assessed the quality of evidence using the GRADE approach.

**Results:**

Twenty articles reporting on 19 studies met our inclusion criteria. All of these studies were conducted in high-income countries and examined different types of interactions, including detailing, industry-funded continuing medical education, and receiving free gifts. While all included studies assessed prescribing behaviors, four studies also assessed financial outcomes, one assessed physicians’ knowledge, and one assessed their beliefs. None of the studies assessed clinical outcomes. Out of the 19 studies, 15 found a consistent association between interactions promoting a medication, and inappropriately increased prescribing rates, lower prescribing quality, and/or increased prescribing costs. The remaining four studies found both associations and lack of significant associations for the different types of exposures and drugs examined in the studies. A meta-analysis of six of these studies found a statistically significant association between exposure and physicians’ prescribing behaviors (OR = 2.52; 95% CI 1.82–3.50). The quality of evidence was downgraded to moderate for risk of bias and inconsistency. Sensitivity analysis excluding studies at high risk of bias did not substantially change these results. A subgroup analysis did not find a difference by type of exposure.

**Conclusion:**

There is moderate quality evidence that physicians’ interactions with pharmaceutical companies are associated with their prescribing patterns and quality.

## Introduction

Promotional activities within pharmaceutical industries were relatively high in the past few years. In 2012, the pharmaceutical industry in the USA spent more than US$27 billion on drug promotion [1]. In Canada, promotional activities were estimated to cost US$30000 per physician per year [2]. Pharmaceutical companies appear to spend much more on promotion than they do on research and development (R&D) [3]. For example, a study based on annual reports of pharmaceutical companies found that ten of the largest global pharmaceutical companies spent a total of US$739 billion on ‘marketing and administration’ between 1996 and 2005 compared to US$288 billion on R&D for the same period [3].

The industry claims that the promotional activities aim to provide health care professionals with scientific and educational information [4]. Also, surveys suggest that many physicians believe that marketing does not influence their prescribing habits or acknowledge that it may have an influence on some physicians but not on themselves [5–7]. Despite these claims, there is evidence suggesting that the interaction of pharmaceutical companies with physicians may have a negative effect on their clinical practice [8–11].

The last identified systematic review assessing the interaction of pharmaceutical companies with physicians was published by Spurling et al. in 2010. The population under study included both physicians in practice and residents in training [11]. The review found high degrees of heterogeneity that may have been due to the diverse populations under study (i.e., both practicing and in-training physicians). The review by Spurling et al. could not reach definitive conclusions about the degree to which information from pharmaceutical companies decreases, increases or has no effect on the quality, cost or frequency of prescribing.

Since the publication of Spurling’s review, at least eight original studies have been published [12–19]. One of these was a large study of the association between physicians’ receipt of meals from industry and the rates of prescribing the promoted drug to Medicare patients [15]. That study appears to be at lower risk of bias than the previously published studies, and thus would contribute the improving the quality of the evidence. Therefore, the objective of our study was to systematically review the association between physicians’ interactions with pharmaceutical companies and their clinical practice.

## Methods

### Protocol

We followed a detailed methodology that we describe in the protocol included in S1 Appendix. The review followed the Preferred Reporting Items for Systematic Reviews and Meta-Analyses (PRISMA) guidelines (S2 Appendix).

### Eligibility criteria

The inclusion criteria were:

- Type of study design: observational design (e.g., cohort, time series analysis, before-after design, case control, cross sectional), and experimental design (non-randomized controlled trials, and randomized controlled trials);
- Type of participants: practicing physicians as defined in the primary studies
- Type of exposure: targeted interaction between physicians and pharmaceutical companies, where there is direct interaction with the physician. Direct interactions could include individual invitation to a continuing medical education (CME) event; active presentation of industry-related information to the physician; or provision of gifts to the individual;
- Type of control: either no interaction or a lower level of interaction. The intention was to capture studies that stratified the levels of interaction of physicians with medical representatives, e.g., according to the number of visits within a specified period of time.
- Types of outcomes:
  ○ Knowledge of physicians (e.g., accuracy of knowledge related to a specific medication);
  ○ Attitude of physicians (e.g., perceived influence of information from pharmaceutical company on their behavior);
  ○ Behavior of physicians (e.g., prescribing quality; prescribing quantity/frequency; reliance on pharmaceutical companies for drug information; giving drug sample to patients; submitting a formulary request for a drug made by a specific company);
  ○ Financial outcomes (e.g., patient out of pocket expenses; prescription costs);
  ○ Patients’ clinical outcomes.

The exclusion criteria were:

- Qualitative studies, ecological studies, econometric studies, editorials, letters to the editor, and non-English studies.
- Studies focusing on medical students and physicians in training.
- Studies assessing the relationship between attitudes and behavior
- Studies assessing non-targeted interactions (e.g., journal advertisement) and research funding
- Studies assessing interventions to share industry-independent drug information or interventions to reduce interactions between physicians and pharmaceutical companies. The latter has been addressed in a recent systematic review [20].

We did not exclude studies based on date of publication. We did not exclude any study based on risk of bias. Instead, we conducted sensitivity analyses excluding studies at high risk of bias, and also took risk of bias into account when grading the quality of evidence using GRADE approach.

### Search strategy

We used OVID interface to electronically search MEDLINE and EMBASE in July 2016. The search included both free text words and medical subject headings. It combined terms for physicians and pharmaceutical industry and did not use any search filter. A medical librarian assisted with designing the search strategy (see supporting file S3 Appendix for full search strategy). In addition, we reviewed the references lists of included and relevant primary studies and literature reviews.

### Selection of studies

Two reviewers screened the title and abstracts of identified citations for potential eligibility in duplicate and independently. We retrieved the full text for citations considered potentially eligible by at least one of the two reviewers. The two reviewers then screened the full texts in duplicate and independently for eligibility. The reviewers resolved disagreement by discussion or with the help of a third reviewer. They conducted calibration exercises and used a standardized and pilot tested screening form.

### Data collection

Two reviewers abstracted data from eligible studies using a standardized and pilot tested screening form with detailed written instructions. When needed, disagreement was resolved with the help of a third reviewer. The data abstracted included: type of study, funding source, characteristics of the population, exposure, outcomes assessed, and statistical data. For studies including both attending physicians and residents, we attempted to contact authors for data relating to the former group.

### Assessment of risk of bias in included studies

Two reviewers assessed the risk of bias in each eligible study in duplicate and independently. They resolved disagreements by discussion or with the help of a third reviewer. We used the tool suggested by the GRADE working group for assessing the risk of bias for observational studies [21].

We calculated the risk of bias using the following criteria:

- Failure to develop and apply appropriate eligibility criteria (e.g., no clear eligibility criteria, convenient sampling, under- or over-matching in case-control studies, selection of exposed and unexposed in cohort studies from different populations, and low response rate (<60%) with no attempts to compare non-respondents to respondents) [22].
- Flawed measurement of exposure (e.g., differences in measurement of exposure such as recall bias in case- control studies, and subjective or self-reported assessment of exposure)
- Flawed measurement of outcome (e.g., differential surveillance for outcome in exposed and unexposed in cohort studies, and subjective or self-reported assessment of outcome)
- Failure to adequately control confounding (e.g., failure of accurate measurement of all known prognostic factors, absence of control group in a before-after study, failure to match for prognostic factors and/or adjustment in statistical analysis
- Incomplete follow-up or failure to control for loss-to-follow up

We graded each potential source of bias as high, low or unclear risk of bias. We used unclear when the authors did not report enough information for us to make the judgment.

### Data synthesis

We calculated the kappa statistic to assess the agreement between reviewers for full text screening.

We conducted a meta-analysis to pool the results across studies for the association between ‘targeted interactions with physicians’ as the exposure of interest, and ‘changes in physician prescribing behavior’ as the outcome of interest. We contacted the authors of studies that appeared to have measured the outcome and exposure of interest, but did not report data (such as odds ratio or standard error) that we could include directly. We received responses from authors of 7 out of 9 relevant studies. The authors provided us with the needed information for only two out of the seven studies. Please refer to S1 Table for a summary of author contacts.

We used the following a priori plan for choosing which data to include in the meta-analysis:

- For studies reporting on more than one type of exposure (e.g., gifts, detailing), we treated each exposure as a separate unit of analysis.
- For studies measuring the same outcome at several points in time, we chose the first time point to avoid any potential confounding effects from subsequent measures.
- For studies assessing the association of interest for more than one drug (i.e., reporting more than one association), we included the value that is the closest to the mean of all reported values amongst those associations.

We used the generic inverse variance technique with a random-effects model to pool the association measures across included studies that reported the needed statistical data. We carried out statistical analysis using RevMan (version 5.2).

To take into account the heterogeneity introduced by the different types of exposures (i.e., gifts, detailing, and CME), we stratified the meta-analyses by type of exposure. We tested the results for homogeneity using the I2 test and considered heterogeneity present if I2 exceeded 50%.

In addition, we conducted three post-hoc sensitivity analyses by respectively excluding:

- Studies at high risk of bias;
- Studies funded by pharmaceutical industry;
- Studies measuring the outcome of interest as ‘changes in generic prescription’ or ‘formulary request’ (as these were considered indirect measures compared with the ‘changes in the prescribing of promoted drug’).

Although we had planned to construct funnel plots to assess publication bias, the number of included studies in the meta-analyses was too low to allow for that. Indeed, funnel plots are encouraged for interventions that include at least 10 studies, with a substantially higher number required if significant heterogeneity is present.[23].

We used the GRADE approach to assess the quality of the body of evidence [21]. The GRADE methodology involves rating the initial quality of evidence for an association as high (with observational data), followed by downgrading based on five criteria (risk of bias, inconsistency, imprecision, indirectness and publication bias), and upgrading based on three criteria (large effect size, dose-response gradient, and plausible confounding) [24].

We narratively reported any additional results that we were not able to include in the meta-analysis from eligible studies (this includes studies that could have contributed data to the meta-analysis). Whenever provided, we included the p-value to denote significance of results.

## Results

### Selection of studies

**Fig 1:**
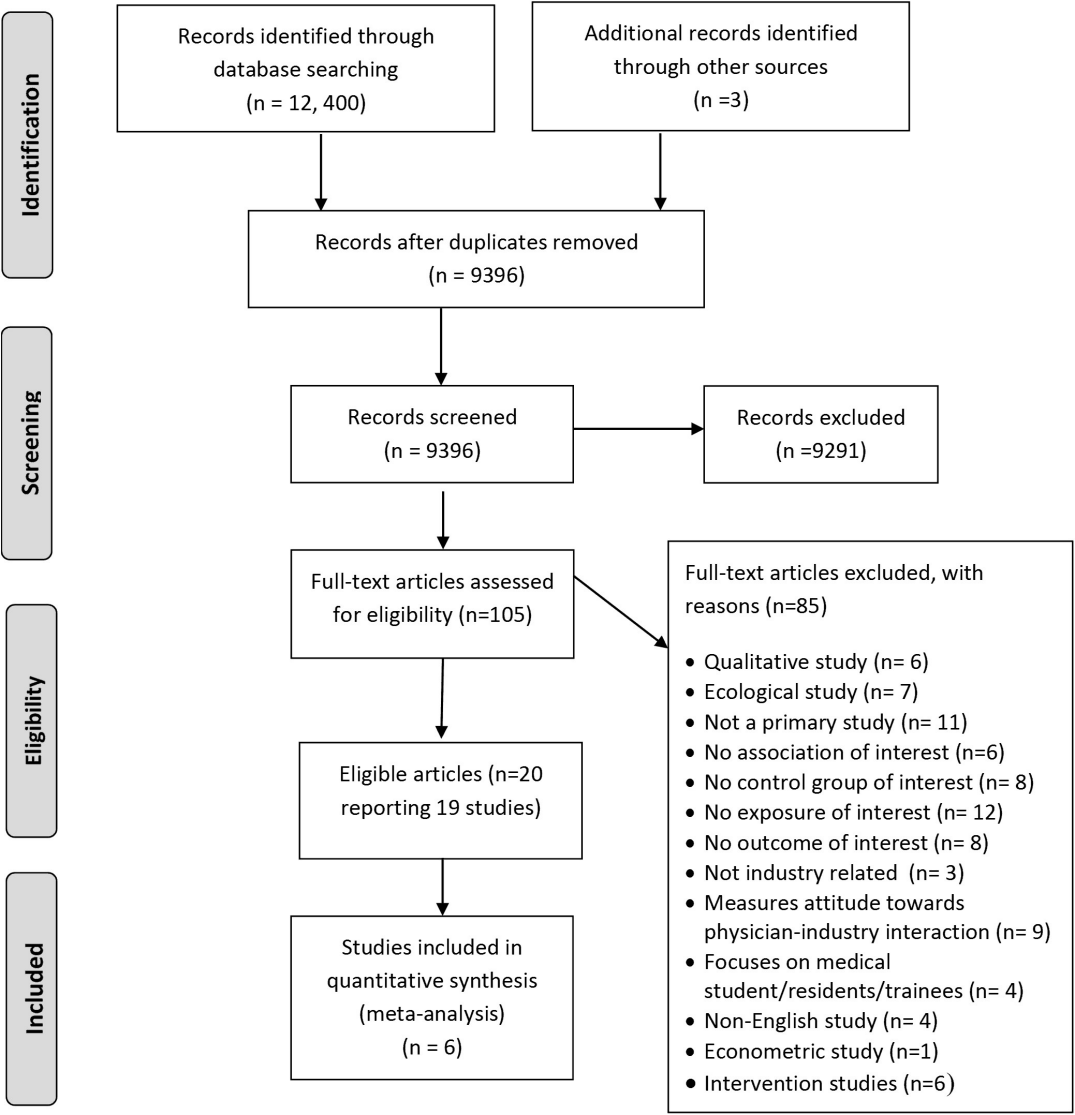
Study flowchart showing the selection process.

Fig 1 shows the study flow. Of the 12, 400 article titles identified by the electronic literature search, 20 articles reporting on 19 studies met our inclusion criteria (two articles reported on different outcomes for the same study) [25, 26]. A list of the excluded studies along with reasons for exclusion is provided in S2 Table. The kappa statistic value for full text screening was 0.89, suggesting high levels of agreement.

### Characteristics of included studies

Table 1 shows the characteristics of the 19 included studies. The design for the majority of studies was cross-sectional (n = 13). Four studies reported using pre-post study designs, [17, 27–29] one was a retrospective cohort study [12] and one was a nested case-control study [30]. The sample sizes in these studies varied between 10 and 279, 669 with a median of 206. One study did not provide adequate information on the exact sample size [18]. The included studies were conducted in the USA (n = 13), Australia (n = 1), Spain (1), Denmark (n = 1), Germany (n = 1) and the Netherlands (n = 2). The publication year ranged from 1972 to 2016. The specialties of the physicians in the majority of studies were primary care (including general practitioners, family medicine, internal medicine) (n = 8); obstetrics and gynecology (OB/GYN) (n = 1); dermatologists (n = 1); mix (n = 3); and unclear (n = 6). The types of exposure assessed were sales representatives’ visits or detailing (n = 10), industry-funded continuing medical education including travel funds (n = 4), and receiving free gifts (e.g. drug samples, meals, gifts in the form of office stationery, and grants and payments) (n = 11). One study assessed a mix of exposures without reporting data specific to each exposure [31]. The types of outcomes assessed were physicians’ prescribing behaviors (n = 19), physicians’ beliefs (n = 1), physicians’ knowledge (n = 1) and financial outcomes (n = 4). Physicians’ prescribing behavior was defined as the changes in quantity or quality of prescriptions. None of the studies assessed clinical outcomes.

**Table 1:** Characteristics of included studies.

Study Name	Study Design	Participants	Exposure	Control	Outcomes
Becker 1972 [34]	Cross sectional survey with self-administered questionnaire	29 general practitioners, 5 osteopaths and 3 internists in a mid-Atlantic state	Use of detail men as sources of prescribing information concerning new drugs.	No use of detail men as sources of prescribing information for new drugs.	**Behavior**: appropriateness and prescribing pattern of physicians.
Haayer, 1982 [31]	A cross-sectional study; self-administered questionnaire in September 1979,	118 general practitioners in Twente, Netherlands	A mix of exposures: “manufacturer’s representatives, drug companies’ mailings, use of samples, drug companies’ journals, drug firm meetings”	Lower levels of exposure	**Behavior**: rational prescribing of physicians based on their responses to eight common-practice case-histories
Bowman, 1988 [27]	A pre-post survey study	197 physician participants, with 121 (61.4%) returning matched forms (course III); setting not clear	Attendance of continuing medical education courses, subsidized heavily by a single but different drug company	Period prior to attending course III	**Behavior:** changes in rates of prescribing by physicians of course-related drugs (immediately prior to six months after each course)
Peay, 1988 [37]	A cross-sectional survey between October and December 1981	124 specialists and general practitioners in private practice in one (or both) of two geographical areas in the vicinity of a major Australian city	• Contact with detail men regarding the drug • Receiving a sample of the drug	Lower levels of exposure	• **Behavior**: prescription of temazepam routinely in preference to alternatives, the timing of prescription of temazepam • **Knowledge**: awareness of physician about the drug being promoted
Orlowski, 1992 [29]	Pre-post study (Data collected between 1987 and 1989)	Physicians from one major medical center (1006 bed); no further information provided. (10 physicians attended the symposium on drug A and 10 attended symposium on drug B)	All-expenses-paid trip to an attractive resort for the physician and a significant other to attend a symposium on one of the company’s drugs. One symposium was on drug A, a new intravenous antibiotic, and another was on drug B, a new intravenous cardiovascular drug.	• Control 1: physicians prior to attending the symposia • Control 2: hospitals with more than 500 beds and major medical centers that used the two drugs but were not exposed to symposium	**Behavior**: physician prescribing patterns of two drugs (approximately 22 months) before and (approximately 17 months) after the expense-paid trips.
Chren, 1994 [30]	Nested case-control study	120 full-time attending physicians at the University Hospitals of Cleveland (874 bed); 105 participated (36 cases and 69 controls)	• Pharmaceutical sales representative visits; • Acceptance of money to attend symposia; • Acceptance of money to speak at symposia	No exposure	**Behavior**: Physicians’ formulary request for a drug made by a specific company
Figueiras 2000 and Caamano 2002[25, 26]	A cross sectional study (Data collected using a 60-item self-administered mail questionnaire. The first questionnaire was mailed out in October, 1992)	405 primary care physicians in Galicia, Northwest Spain; of those 234 physicians responded	• Utilization of information obtained from visiting sales representatives measured with a dichotomous yes/no variable • Number of sale representatives’ visits per week	• Lower levels of exposure (sale representatives’ visits per week) • No utilization of information obtained from visiting sales representatives measured	• **Behavior**: quality of drug prescribed, reflected via 3 indicators combined to produce a global indicator variable. Indicators included: “% of drugs prescribed with no controlled trials for their efficacies; % of drugs prescribed of therapeutic use not elevated in PC; and % of drugs not included in the 1992 formulary for PC “ • **Financial outcome**: expenditure by physician
Mizik, 2004[35]	“Pooled time series cross-sectional study” covering a 24-month period	Panel data comprising 74,075 individual physicians	Sales representative visits per month Receiving free drug samples (per month)	Lower levels of exposure	**Behavior**: number of new prescriptions issued by a physician per month for three studied drugs
Muijrers, 2005[36]	A cross-sectional survey (Data collected using a questionnaire in 2001)	324 GPs in solo practices in the south of the Netherlands (out of 1434 GPs included in an initial survey; those in non-solo practices were excluded)	Visits by pharmaceutical industry representatives measured as the number of visits per month.	Lower levels of exposure	**Behavior**: quality of prescribing measured as ‘adherence to guidelines’ calculated as a weighted average score on 20 prescribing indicators based on general practice guidelines of the Dutch College of General Practitioners.
Symm, 2006[32]	A cross-sectional design with retrospective review of prescription claims data.	23 family physicians providing patient care in three Scott and White regional clinics (8 in exposure group and 15 in control group)	One clinic (clinic X) where sample medications were dispensed	Two clinics (clinics Y and Z) that do not dispense free sample medications	• **Behavior**: number of prescriptions written for study medications in relation to the distribution of free sample medications • **Financial outcome**: average 30-day prescription costs
Miller, 2008[28]	A retrospective study, pre-post removal of drug sample closet (Pre post periods were each for 9 months: March–November 2000 and January–September 2001)	Ambulatory internal medicine practice affiliated with the Wake Forest University School of Medicine and comprising of 10 attending physicians (63 internal medicine residents not eligible for this review)	Prior to the discontinuation of a drug sample closet with samples delivered by pharmaceutical representatives.	After the discontinuation of the drug sample closet	**Behavior**: percentage of medications prescribed to uninsured or Medicaid patients as generics
Anderson 2009[33]	A cross sectional survey study (Data collected between 2006 and 2007)	515 ob-gyns members of the American College of Obstetricians and Gynecologists’ Collaborative Ambulatory Research Network invited; 251 participated	Interaction with sales representatives measured as the reported frequency of eating food provided by a pharmaceutical representative.	Lower levels of exposure	**Behavior**: reported reliance on sales representatives when deciding whether to prescribe a new drug
Søndergaard, 2009[17]	Retrospective cohort study; pre-post design (Data collected between April 2001 and July 2003)	165 general practices in Funen County, Denmark encompassing 273 GPs with 54 080 patients treated with asthma drugs	Promotional visits by pharmaceutical representatives’ promoting a fixed combination of inhaled corticosteroid and long-acting b2-agonist)	Period prior to the first pharmaceutical representative visit	**Behavior**: proportion of dispensings of the promoted drug among all dispensings of fixed combinations of inhaled corticosteroid and long-acting b2-agonists
Pinckney, 2011[19]	Cross-sectional survey study (Data collected using a mailed survey in September 2007)	631 primary care prescribers in the state of Vermont; 206 (or 35%) prescribers returned the survey and met the eligibility criteria	Prescribers with medication samples in their clinics	Prescribers without medication samples in their clinics	• **Behavior**: prescription preference of physicians in response to two clinical vignettes • **Beliefs**: agreeing with statements about sample use”
Pedan 2011[12]	A retrospective cohort study (Data collected between January 2008 and December 2009)	751 physicians who participated in the Metropolitan Area Promotional Audit and received at least 6 sales calls for Lipitor, Vytorin, or Crestor and who had written at least 10 new prescriptions for any of these drugs in the same time period	Sales calls (including detailing, sample drops and meals)	Lower levels of exposure	**Behavior**: total monthly number of new prescriptions (NRx) written by a physician for the three leading statin brands
Lieb, 2014[14]	Cross-sectional study (Data collected via an online survey from start of the 3rd quarter of 2010 to end of the 2nd quarter of 2011)	160 German doctors (131 GPs/internal medicine specialists, 26 psychiatrists or neurologists and 3 cardiologists)	• Visit by pharmaceutical sales representatives during last 12 months • Acceptance of gifts in form of office stationary offered by sales representatives	Lower level of exposure (pharmaceutical sales representative visits per week, frequency of accepted gifts)	• **Behavior**: individual prescribing data of physicians over a year for all on-patent branded, off-patent branded, and generic drugs prescription • **Financial outcomes**: total expenditure
Hurley, 2014[18]	Cross-sectional study (Primary initial diagnosis of acne vulgaris or rosacea in 2010)	• Offices of nationally representative US dermatologists from the National Disease and Therapeutic Index • Dermatologists in an academic medical center clinic	Offices of nationally representative dermatologists where free samples for acne are often available	An academic medical center clinic without samples (where free drug samples have been banned since 2004)	• **Behavior**: brand-name medications prescribed per office visit for a primary initial diagnosis of acne vulgaris or rosacea in 2010 • **Financial outcomes**: mean cost of acne medications prescribed per office visit.
Dejong 2016 [15]	Cross-sectional study (Data collected from August 2015 to December 2015)	279 669 physicians who wrote Medicare prescriptions in any of 4 selected drug classes	Receipt of an industry-sponsored meal promoting the drug of interest.	Physicians receiving no target meals	**Behavior**: prescribing rates of promoted drugs compared with alternatives in the same class
Yeh 2016[16]	Cross-sectional study (Data collected from January 1 to December 31, 2011)	All 2444 physicians in Massachusetts who wrote prescriptions for statins paid for under the Medicare drug benefit in 2011.	Industry payment to physician (: food, grants or educational gifts, bona fide services, educational training,)	No industry payment	**Behavior**: rate of prescribing brand-name statins as compared with generic statins for lowering cholesterol.

### Risk of bias

**Fig 2:**
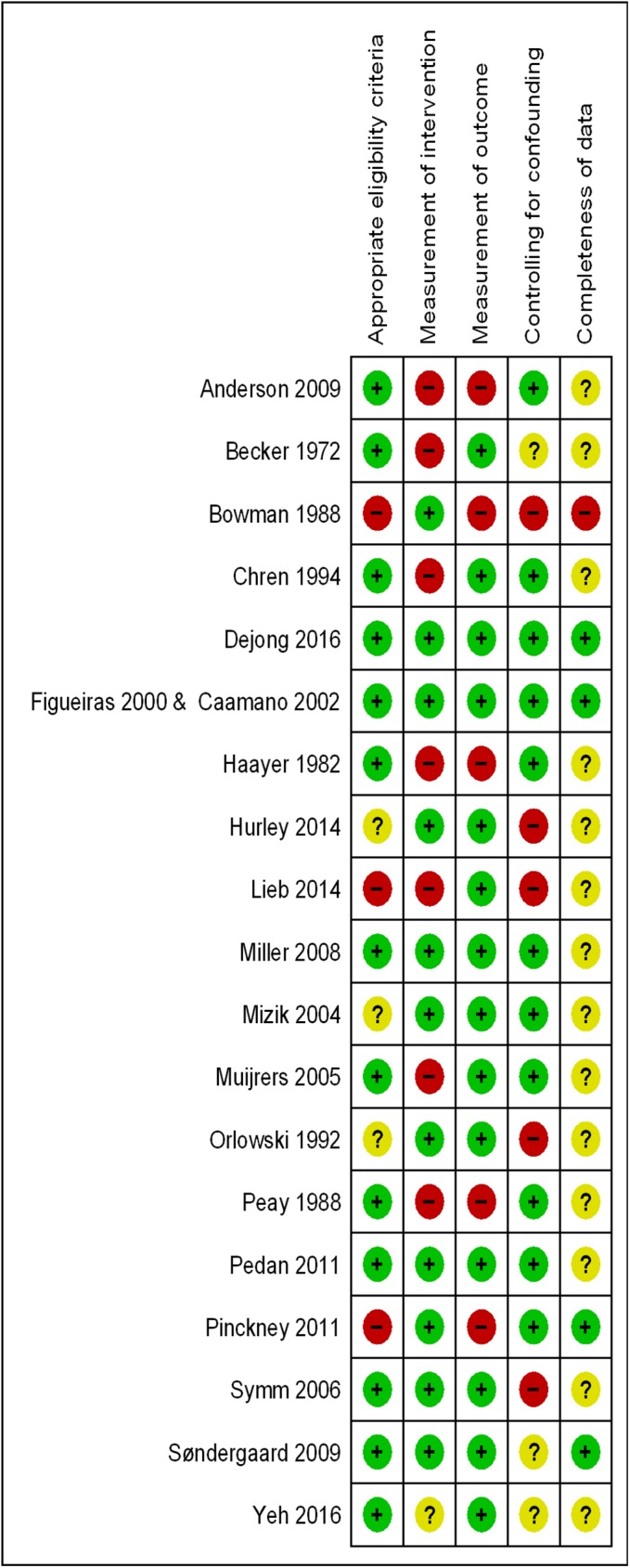
Risk of bias summary reflecting reviewers’ judgments about each risk of bias item for included studies.

The detailed judgments about each risk of bias item for included studies are displayed in Table 2. Fig 2 shows the corresponding risk of bias summary for these studies. For the majority of the studies, the risk of bias was judged to be low for ‘appropriate eligibility criteria’, ‘measurement of intervention’, and ‘measurement of outcome’, except for the ‘completeness of data’ that was judged as unclear. For ‘controlling for confounding’ the risk of bias was judged as low for nine studies, unclear for four studies, and high for six studies.

**Table 2:** Risk of bias and funding source for each included study.

Study Name	Funding source	Developing and applying appropriate eligibility criteria	Measurement of exposure	Measurement of outcome	Controlling for confounding	Completeness of data
Becker 1972[34]	Grants from the National Center for Health Services Research and Development USPHS &from National Institute of Health DHEW	Low: All actively practicing primary physicians in the county included with a response rate of 84% (37 out of 44)	High: Self-reported scale to collect data on physician’s use of detail men as sources of prescribing information.	**Low** for prescription pattern outcome (computer-generated profiles of actual prescribing of chloramphenicol); **High** for prescription appropriateness (Two panels of experts evaluated physician's general prescribing behavior relative to five common complaints and five common illnesses)	Unclear: Controlled for variance in numbers and types of patients.	**Unclear**: Author did not comment on completeness of data
Haayer, 1982[31]	Ziekenfondsraad (Health Insurance Fund)	**Low:** Population-based study targeting all GPs (148) in Twente, resulting in a final list of 131 GPs (118 GPs agreed to participate)	**High**: Study interview pilot tested but not validated. Risk of recall bias and/or social desirability bias since it is self-reported by physician	**High**: Study questionnaire pilot tested but not validated. A panel of experts rated the rationality of prescribing on four different scales that have then been combined.	**Low**: Authors conducted stepwise multiple regression to adjust for potential confounders.	**Unclear**: Author did not comment on completeness of data
Bowman 1988[27]	Not reported	**High:** Sampling method not explained; low response rate (49%); characteristics of non-responders and responders were not compared	**Low:** No reason to suspect that measurement of attendance of course was not valid	**High**: Non validated self-report survey was used.	**High**: Control for confounding variables not reported (Analyses did not take into account other predictors of prescription behavior)	**High:** Response rate for different courses varied between 43% and 76%)
Peay, 1988[37]	Australian Research Grants Scheme and the Flinders University Research Budget	**Low**: Clear sample selection and eligibility criteria with 60% response rate.	**High**: Survey/interview method used to measure the exposure. Authors did not report on validity and reliability of the interview guide.	**High:** No objective measurement of outcome (survey/interview method used to measure behavior. Also, authors did not report on the validity and reliability of the interview guide)	**Low**: Additional multivariate analyses were carried out.	**Unclear:** The author did not mention any missing data
Orlowski, 1992[29]	Not reported	**Unclear:** No clearly defined eligibility criteria or sampling method (physicians who had accepted invitations to attend symposia were identified by general questioning of colleagues and were affiliated with one institution)	**Low:** No reason to suspect that measurement of attendance of symposia was not valid	**Low**: Objective measurement of outcome; prescribing pattern was tracked retrospectively using the hospital pharmacy inventory usage reports; both drugs were used only in hospitalized physicians; National usage data for the 2 drugs was obtained from Pharmaceutical Data Services	**High:** Study focuses on the symposia and ignores the impact of other approaches to marketing including advertisements, salesman contacts, and journal articles. Also, analysis did not adjust for the fact that the second course was offered approximately 20 months after drug B had been added to the hospital formulary	**Unclear:** Author did not comment on completeness of data
Chren, 1994	National Institute of Arthritis, USA; Skin Diseases Research Center and Clinical Analysis Project, University Hospitals of Cleveland,	**Low:** Clear sample selection and eligibility criteria. Response rate was 88%	**High:** Survey pilot tested but not validated. Risk of recall bias and/or social desirability bias since it is self-reported by physician	**Low**: Objective measurement of outcome by reviewing the standard formulary request forms	**Low:** Multivariable logistic regression models controlled for physician age, gender, departmental appointment, and number of patients seen per week	**Unclear:** The author did not mention any missing data
Figueiras 2000 and Caamano, 2002[25, 26]	Spanish Ministry of Health and Consumption	**Low:** Clear eligibility criteria with random selection of subjects. Exposed and control subjects were from the same population. Response rate was 75%	**Low**: A “valid and reliable” self-administered mailed questionnaire was used to collect information on exposure	**Low**: Objective measurement of outcome using “the database of the accounting archives of the National Health Service, which includes all prescriptions served in all the pharmacies in Galicia.”	**Low:** Analyses controlled for confounding variables such as type of practice, number of identification cards, number of patients seen per day, the service accessibility\ for the patients, unemployment rate and population distribution.	**Low:** Missing data were controlled for by carrying out multiple imputation
Mizik, 2004[35]	Institute for the Study of Business Markets (ISBM) at Pennsylvania State University	**Unclear:** No clearly defined eligibility criteria or sampling method	**Low**: Objective measurement of exposure using panel data from US pharmaceutical manufacturer	**Low:** Objective measurement of outcome using panel data from a U.S. pharmaceutical manufacturer	**Low:** Dynamic fixed-effects distributed lag regression model controlled for a range of potential confounding factors	**Unclear:** Author did not mention any missing data
Muijrers, 2005[36]	Dutch Pharmacist’s Association and the CZ health insurance company	**Low:** Clear selection criteria. Studied general practitioners in south of the Netherland. Response rate was 71%.	**High:** Self-reported survey used to measure exposure. Authors did not report on validity and reliability of survey tool.	**Low:** Objective measurement of prescribing indicators using a prescription database compiled by linking pharmacy databases from 379 pharmacies.	**Low:** A multiple regression analysis included the a range of predictors	**Unclear:** Author did not mention any missing data
Symm, 2006[32]	Scott & White Institutional Research Fund	**Low:** Clear selection criteria. The 25 sample medications selected comprised 84% of samples dispensed during study period	**Low:** Objective measurement of exposure using the 2003 sample logs which were reported to be 95% to 100% accurate	**Low:** Objective measurement of outcome using Scott & White Health Plan prescription claims data.	**High:** Although case-mix adjustment data indicates very similar practices among the 3 clinics, “there may still have been differences that we overlooked or were unable to measure”	**Unclear:** Author did not mention any missing data
Miller, 2008[28]	Not reported	**Low:** Clear eligibility criteria with all 10 attending physicians in a large resident-faculty practice selected.	**Low:** Objective measurement of exposure (observation of existing sample cabinet being discontinued)	**Low:** “Presence of an electronic pharmacy database allowed to abstract accurate data for a wide assortment of medications prescribed by a variety of physicians.”	**Low:** Controlled for a range of potential confounders. Also conducted sensitivity analysis which did not significantly affect results	**Unclear:** Author did not mention any missing data
Anderson 2009[33]	Office of Medical Applications of Research, National Institutes of Health, and Maternal and Child Health Bureau, Health Resources and Services Administration, USA	**Low:** Random selection of participants from a nationally representative database. Response rate of 49%; however, factors for which responders and non-responders differed were not associated with industry attitudes and interaction	**High:** Study questionnaire pilot tested but not validated. Risk of recall bias and/or social desirability bias since it is self-reported by physician	**High:** Study questionnaire pilot tested but not validated. Risk of recall bias and/or social desirability bias since it is self-reported by physician	**Low:** Authors constructed three linear regression models controlling for: reading guidelines on physician-pharma interactions, physician characteristics, physician practice, physician perceived value of industry drug information.	**Unclear:** Authors did not comment on completeness of data
Søndergaard, 2009[17]	AstraZeneca funded the study through a grant to the Research Unit for General Practice in Odense	**Low:** Population-based study targeting all GPs (191) in the county, resulting in a final list of 165 GPs	**Low**: Objective measurement of exposure using AstraZeneca’s database	**Low**: “Outcome data based on a highly valid and complete register covering all prescribed asthma drugs”	**Unclear**: While authors controlled for calendar time and device preferences, they did not control for other factors such as competing firm’s drug marketing effort, which can also affect drug preference.	**Low:** Only two of the requested to be withdrawn from the analysis
Pinckney, 2011[19]	Freeman Medical Scholars Program, The Champlain Valley Area Health Education Center, and the Attorney General Consumer and Prescriber Grant Program	**High:** Low response rate was low (35% of all 631 primary care clinicians practicing in the state of Vermont)	**Low:** Absence or presence of sample closet in clinic measured using “several items from a survey developed and validated by Chew et al”.	**High:** Prescription preference was based on a hypothetical scenario and not actual behavior. Attitude measurement used a scale that was not reported as validated	**Low:** Authors used multivariable regression models to adjust for potential confounders.	**Low:** Exclusion due to incomplete data less than 6%
Pedan 2011[12]	Inventiv Health	**Low:** Clear eligibility criteria. Results were “robust to alternative … sample selection criteria”. The panel is geographically and socioeconomically representative.	**Low:** Objective measurement of exposure using the unique representative dataset.	**Low**: Objective measure of outcome using the dispensing records from a large number of nationwide and regional pharmacy chains (inVentiv Health computerized pharmacy prescription database)	**Low**: Authors accounted for competitive promotions, various physicians, practice settings, patient base, and market dynamic characteristics.	**Unclear:** Authors did not comment on completeness of data
Lieb, 2014[14]	No support or funding to report	**High:** No clear eligibility criteria; Low response rate 11.5% (n = 160)	**High:** No objective measurement of exposure; doctors completed an online questionnaire. Also, discrepancy in categorizing exposure and control group	**Low:** Objective measurement using prescribing data over a year for all on-patent branded, off-patent branded, and generic drugs from the Bavarian Association of Statutory Health Insurance Physicians	**High**: Control for confounding variables not reported “We have not recorded or taken into consideration any other factors that could influence the prescribing habits of doctors and may interact with the PSR visits”	**Unclear:** Authors did not comment on completeness of data
Hurley, 2014[18]	National Heart, Lung, and Blood Institute & National Center for Research Resources and the National Center for Advancing Translational Sciences, National Institutes of Health	**Unclear:** While national data was obtained from the National Disease and Therapeutic Index (NDTI), physicians were selected from master lists of the American Medical Association (AMC) and the American Osteopathic Association through random sampling. Differences in demographics between patients at the AMC and on a national level	**Low:** Objective measurement of outcome using data from a large academic medical center without samples extracted from Stanford University’s Epic electronic database via the Center for Clinical Informatics	**Low:** Objective measurement of outcome using national data obtained from the National Disease and Therapeutic Index (NDTI). Drug prices were directly quoted from customer service representatives of a major pharmacy in July 2013.	**High:** “The observed differences in prescribing habits may be attributed to other forms of pharmaceutical marketing that were not adequately captured in our study, such as the number of visits by or gifts from pharmaceutical representatives or the use of co-payment discount cards, which can also influence prescribing patterns	**Unclear**: Authors did not comment on completeness of data
Dejong 2016 [15]	National Center for Advancing Translational Sciences, National Institutes of Health; and by the Hawaii Medical Service Association Endowed Chair in Health Services and Quality Research at University of Hawaii	**Low:** Clear eligibility criteria. The study population included 279 669 physicians. Of these, 155 849 physicians wrote more than 20 prescriptions in 1 of the 4 target drug classes and were assigned to study groups.	**Low:** Objective measurement of exposure using the 2013 Open Payments database which describes the value and the drug or device being promoted for all payments to physicians from August through December 2013	**Low**: Objective measurement of prescribing data for individual physicians from Medicare Part D	**Low:** Multivariable grouped logistic regression models with binomial physician-level prescribing data, and adjusting for a number of covariates	**Low:** 5% of payments promoting the target drugs were excluded from the regression analysis
Yeh 2016 [16]	Not reported	**Low:** “From 363653 physicians in the Medicare Part D prescription claims database, we identified 9628 with a business address in Massachusetts, of whom 2444 had associated statin prescriptions covered by Medicare.”	**Unclear:** Although exposure was measured using Massachusetts physicians payment database compiled by Massachusetts Department of Health, the authors were unable to determine the frequency of misattribution of the payment category or underreporting of payment	**Low:** Objective measurement of outcome using Part D Medicare prescriptions claims data prepared by the Centers for Medicare and Medicaid Services (CMS).	**Unclear:** Although authors mentioned conducting linear regression models, they stated that they were not able to control for certain physician characteristics (e.g., practice characteristics, level of experience) which may have an impact on prescribing patterns.	**Unclear:** Authors did not mention any missing data

### Findings of studies

Table 3 provides a summary of the outcomes and the statistical results reported for each included study. Out of the 19 included studies, six reported data in a format that could be included in the meta-analysis of the association between the exposure and the behavior (i.e., reported odds ratio or risk ratio or provided raw data allowing the calculation of an odds ratio) [15, 17, 19, 27, 28, 30]. Below, we present the results of those six studies and their meta-analysis. We then narratively report any additional results that we were unable to include in the meta-analysis from eligible studies.

**Table 3:** A summary of the outcomes and statistical results of each included study.

Study Name	Exposure	Outcomes	Statistical results
Becker 1972 [34]	Detailing	**Behavior**: appropriateness and prescribing pattern of physicians.	Use of detail men as sources of prescribing information concerning new drugs was significantly associated with higher prescription of the drug chloramphenicol by primary care physicians (p<0.01), and a poorer rating of prescription quality (p<0.01) by two panels of experts relative to five common complaints and five common illnesses
Haayer, 1982 [31]	A mix of exposure (without reporting data specific to each exposure)	**Behavior**: Rational prescribing of physicians based on their responses to eight common-practice case-histories	Reliance on information from pharmaceutical industry was negatively associated with prescribing rationality (p<0.001)
Bowman, 1988 [27]	Industry- sponsored CME	**Behavior**: changes in rates of prescribing by physicians of course-related drugs	The number of new prescriptions for the sponsoring company’s drug increased statistically from 31.4% to 50.1% pre-post course (p<0.05%)
Peay, 1988 [37]	• Detailing • Receiving free drug	**Behavior**: prescription of Temazepam routinely in preference to alternatives, the timing of prescription of Temazepam	• Physicians who had contact with detailmen regarding Temazepam reported earlier awareness of it (p < 0.041), were more likely to rate it as a moderate advance (as opposed to a minor advance or no advance at all) (p< 0.028), were more likely to have prescribed it (p< 0.0031), reported prescribing it earlier (p< 0.005) and were more likely to prescribe it routinely in preference to alternatives (p< 0.014). • Physicians who had received a sample of Temazepam, compared to those who had not, were more likely to have prescribed it (p < 0.001) and more likely to say that they now usually prescribe it rather than the alternatives (p < 0.006).
Orlowski, 1992[29]	Industry-sponsored CME	**Behavior**: physician prescribing patterns of the two drugs before and after the expense-paid trips	The ‘expense-paid seminar at a resort’ was associated with a significant increase in the prescribing of the promoted drugs within a few months of each symposium compared to their use before the symposium (p<0.001)
Chren, 1994 [30]	• Detailing • Attending industry-sponsored educational symposia • Speaking at industry-sponsored educational symposia	**Behavior**: Physicians’ formulary request for a drug made by a specific company	• Physicians who had met with pharmaceutical representatives were significantly more likely to have requested that drugs manufactured by specific companies be added to the formulary, than other physicians (OR = 3.4; 95% CI: 1.8–6.6). • Increased odds of formulary requests were obtained for physicians who had accepted money from those companies to attend educational symposia (OR = 7.9; 95% CI: 1.1–55.6). • Increased odds of formulary requests were obtained to speak at educational symposia (OR = 3.9; 95% CI: 1.2–12.7).
Figueiras 2000 [26]and Caamano 2002[25]	Detailing	**Behavior**: quality of drug prescribed, reflected via 3 indicators combined to produce a global indicator variable; Utilization of information obtained from visiting sales representatives	• Using information obtained from pharmaceutical representatives is associated with a higher percentage of prescription drug not included in the primary care formulary and with a higher “global indicator variable”, thus reflecting lower prescription quality. • Utilization of the visiting marketers’ information remained significantly associated with higher amounts of prescription (p = 0.048) Utilization of the visiting marketers’ information was significantly associated with higher expenditure per physician (p = 0.035); however number of sales representative visits was not statistically associated with expenditure.
Mizik, 2004 [35]	• Detailing • Receiving free samples	**Behavior**: number of new prescriptions issued by a physician per month for three studied drugs A, B and C	• The estimated total effects of detailing on new prescriptions (average number per month by drug) were: 1.56 (95% CI: 0.80–2.23) for drug A; 0.32 (95% CI: 0.22–0.43) for drug B; and 0.15 (95% CI: 0.11–0.20) for drug C. • Receiving free sample medications had a statistically significant but small impact on the number of new prescriptions issued by a physician per month for three studied drugs
Muijrers, 2005[36]	Detailing	**Behavior**: quality of prescribing measured as ‘adherence to guidelines’	Frequent visits from pharmaceutical industry representatives had a significantly negative correlation with the quality of prescription of general practitioners measured as “adherence to guidelines” (p<0.05)
Symm, 2006[32]	Receiving free samples	• **Behavior**: number of prescriptions written for study medications in relation to the distribution of free sample medications • **Financial outcome**: average 30-day prescription costs	• Family physicians in clinic X significantly wrote the largest proportion of prescriptions for study medications (p <0.0001) versus non-study medications; significantly prescribed the lowest proportion of preferred name brand medications (p <0.0001); • Family physicians in clinic X were significantly higher in the average cost per 30-day prescription than those in clinic Y or Z. The average cost of a 30-day prescription differed significantly by clinic (p <0.0001).
Miller, 2008 [28]	Receiving free samples	**Behavior**: % of medications prescribed to uninsured or Medicaid patients as generics.	The absence of the sample closet was associated with uninsured patients as well Medicaid patients receiving a generic prescription (OR = 4.54; 95% CI: 1.37–15.0)
Anderson 2009 [33]	Industry meal	**Behavior**: reported reliance on sales representatives when deciding whether to prescribe a new drug	Frequency of eating industry-funded food was associated with greater reliance of OB/GYNs on pharmaceutical representatives for drug information when prescribing new medications: first regression analysis (β = 0.16, 95% CI: 0.02–0.31)
Søndergaard, 2009 [17]	Detailing	**Behavior**: proportion of dispensings of promoted drug among all dispensings of fixed combinations of inhaled corticosteroid and long-acting b2-agonists.	Effect of the first visit of drug representatives on the general practitioner’s drug preference favoring the marketed drug (odds ratio (OR): 2.39; 95% CI: 1.72–3.32).
Pinckney, 2011[19]	Receiving free samples	• **Behavior**: prescription preference of physicians in response to two clinical vignettes • **Beliefs**: agreeing with statements about sample use, measured on 5-point Likert scales, subsequently dichotomized into “Agree” versus “Don’t agree”	• Clinicians with samples available were less likely to prescribe thiazide diuretics according to clinical practice guidelines [OR = 0.2 (95% CI: 0.06–0.68)]. To test the robustness of this conclusion, authors conducted a full regression which showed that clinicians with samples were still less likely to select a thiazide diuretic [OR = 0.15 (95% CI: 0.04–0.56]. • Prescribers with samples were significantly more likely to believe that samples: are liked by patients, expedite treatment, help patients who cannot afford their medication, reduce patient costs, and help physicians assess the efficacy of medications. Most prescribers with samples still agreed that they alter treatment plans and increase the costs of care
Pedan 2011 [12]	• Detailing • Receiving free samples • Industry meal	**Behavior**: total monthly number of new prescriptions (NRx) written by a physician for the three leading statin brands	• Detailing produced a highly significant positive impact on new prescriptions for Lipitor and Crestor, although results were not significant for Vytorin. • Sample dispensing had significant positive effect for Crestor (p<0.01) and Vytorin (p<0.05). Results were not significant for Lipitor. • Free meals had a significant positive impact on all three statin brands: Lipitor (p<0.05), Crestor (p<0.05) and Vytorin (p<0.01)
Lieb, 2014 [14]	• Detailing • Industry-sponsored CME • Receiving gifts in the form of office stationery	• **Behavior**: individual prescribing data of physicians over a year for all on-patent branded, off-patent branded, and generic drugs prescription • **Financial outcome**: lower expenditure on off-patent	• Frequently visited practices had a significantly higher number of prescriptions and total daily doses per patient compared to more rarely visited practices (the results were no longer significant after taking into consideration the number of patients per office). • Compared to doctors who frequently, occasionally or rarely took part in sponsored CME events, doctors who mentioned that they never took part in such events had a lower number of on patent-branded drug prescriptions per patient (mean ± SD; 1.05±0.35 vs. 1.27±0.55; p = 0.005, a higher proportion of generics (83.28±7.77% vs. 76.34±13.58%; p<0.0005) and lower expenditure on off-patent branded drugs per patient (£27.36±23.23 vs. £43.75±43.22; p = 0.002). • Compared to physicians who only occasionally, rarely or never accepted stationery, physicians who always or frequently accepted gifts in the form of office stationery prescribed higher daily dose totals per patient (mean ± SD; 491.97±158.95 vs. 420.53±140.57; p = 0.003) and more generics (mean ±SD; 385.52±147.52 vs. 319.43±133.69; p = 0.004)
Hurley, 2014[18]	Receiving free drug samples	• **Behavior**: brand-name medications prescribed per office visit for a primary initial diagnosis of acne vulgaris or rosacea in 2010 • **Financial outcomes**: the mean cost of acne medications prescribed per office visit.	• The increase in provision of samples with a prescription by dermatologists was strongly correlated (r = 0.92) to the increase use of branded generic drugs promoted by these samples. For physicians at local academic centers where free samples are prohibited, only 17% of the commonly prescribed medications were for branded or branded generic drugs compared to 79% for office-based dermatologists on a national level where free samples are available. • The national mean total retail cost of prescriptions was conservatively estimated to be twice as higher (roughly $465 nationally versus $200 at an academic medical center where samples were prohibited)
Dejong 2016[15]	Industry meal	**Behavior:** Prescribing rates of promoted drugs compared with alternatives in the same class	Physicians who received a single meal promoting the drug of interest had higher rates of prescribing of rosuvastatin over other statins (odds ratio (OR) = 1.18; 95% CI: 1.17–1.18); nebivolol over other β-blockers (OR = 1.70; 95% CI: 1.69–1.72); olmesartan over other ACE inhibitors and ARBs (OR = 1.52; 95% CI: 1.51–1.53); and desvenlafaxine over other SSRIs and SNRIs (OR = 2.18; 95% CI: 2.13–2.23).
Yeh 2016[16]	Industry payments for different types of gifts (e.g. meal; grants/educational gifts; educational training)	**Behavior:** Rate of prescribing brand-name statins as compared with generic statins for lowering cholesterol	Among physicians with industry payments reported in the Massachusetts database, every $1000 in total payments received was associated with a 0.1% increase in the rate of brand-name statin drug prescribing (95% CI, 0.06%–0.13%; P < .001). Payments for educational training were associated with an average 4.8% increase in brand-name prescribing compared with no receipt of educational training (95%CI, 1.55–7.95;P = .004), but the other payment types were not.

### Results of the meta-analysis

The study design of the six included studies were retrospective (n = 2), nested-case control (n = 1), pre-post (n = 1) and cross-sectional studies (n = 2). These studies assessed the following types of interactions (with some studies reporting on more than one type): detailing (n = 2); industry-funded continuing medical education including travel funding (n = 2); and receiving free gifts (drug samples and meals) (n = 3).

Sondergaard et al. conducted a retrospective cohort study and reported a statistically significant effect of the first visit of drug representatives on the general practitioner’s drug preference favoring the marketed drug (odds ratio (OR) 2.39; 95% confidence interval (CI) 1.72–3.32) [17]. The effect on drug preference increased further after the second visit (OR = 1.51; 95% CI: 1.19–1.93), but no significant change was noted after the third visit (OR = 1.06; 95% CI: 0.94–1.20). We considered the data for the first visits only as the analysis for subsequent visits may be confounded by the effect of the previous visits as highlighted by the authors: “the effect of promotional visits could in part be caused by representatives selecting practices with a higher probability of adopting the promoted drug. Although we have controlled for the time until first visit, a selection effect cannot be excluded.” Also, none of the remaining studies included in the meta-analysis reported data for subsequent visits.

Bowman et al. conducted a pre-post survey showing the effects of attendance of three continuing medical education courses, each subsidized by a single but different drug company, and the changes in rate of prescribing by physicians of course-related drugs [27]. We excluded the results for courses I and II, given data were not matched. In course III, Diltiazem was the sponsoring company's drug. Prescribing Diltiazem most frequently to new patients statistically increased from 22.3% to 33.9% pre-post course (p<0.05). In addition, the number of new prescriptions for Diltiazem increased statistically from 31.4% to 50.1% pre-post course (p<0.05%) [27]. We considered the first outcome and conducted sensitivity analysis that demonstrated no important changes in results.

Pinckney et al. examined in a cross-sectional study the interaction between the availability of medication samples in the clinics and the prescription preference of primary care prescribers (stated as the name of the medication) in response to two clinical vignettes [19]. Clinicians who did not have samples in their offices were more likely to prescribe hypertension medication according to clinical practice guidelines (p<0.01), and more likely to prescribe a depression medication that was generic (p = 0.02) [19]. Multivariable regression models were conducted only for the hypertension vignette. The findings showed that clinicians with samples were still less likely to select thiazide diuretic that is the preferred treatment for hypertension (OR = 0.15; 95% CI: 0.04–0.56).

Miller et al. conducted a retrospective study to look at the association of a pre-post removal of drug sample closet and the percentage of medications prescribed to uninsured or Medicaid patients as generics. Following a logistic regression model, the authors found that the absence of the sample closet was associated with uninsured patients receiving a generic prescription (OR = 4.54; 95% CI: 1.37–15.0) [28].

In a nested case-control study, Chren el at found that physicians who had met with pharmaceutical representatives were significantly more likely to have requested that drugs manufactured by specific companies be added to the formulary, than other physicians (OR = 3.4; 95% CI 1.8–6.6) [30]. Similar increased odds of formulary requests were obtained for physicians who had accepted money from those companies to attend educational symposia (OR = 7.9; 95% CI: 1.1–55.6), or to speak at educational symposia (OR = 3.9; 95% CI: 1.2–12.7). We treated each exposure as a separate unit of analysis in the meta-analysis.

Dejong et al. examined in a cross-sectional study the association between the receipt of an industry-sponsored meal promoting the drug of interest and prescribing rates of promoted drugs compared with alternatives in the same class [15]. Physicians who received a single meal promoting the drug of interest had higher rates of prescribing Rosuvastatin over other statins (OR = 1.18; 95% CI: 1.17–1.18), Olmesartan over other ACE inhibitors and ARBs (OR = 1.52; 95% CI: 1.51–1.53), Nebivolol over other β-blockers (OR = 1.70; 95% CI: 1.69–1.72), and Desvenlafaxine over other SSRIs and SNRIs (OR = 2.18; 95% CI: 2.13–2.23). We included the value that is the closest to the mean of all reported values amongst those associations.

**Fig 3:**
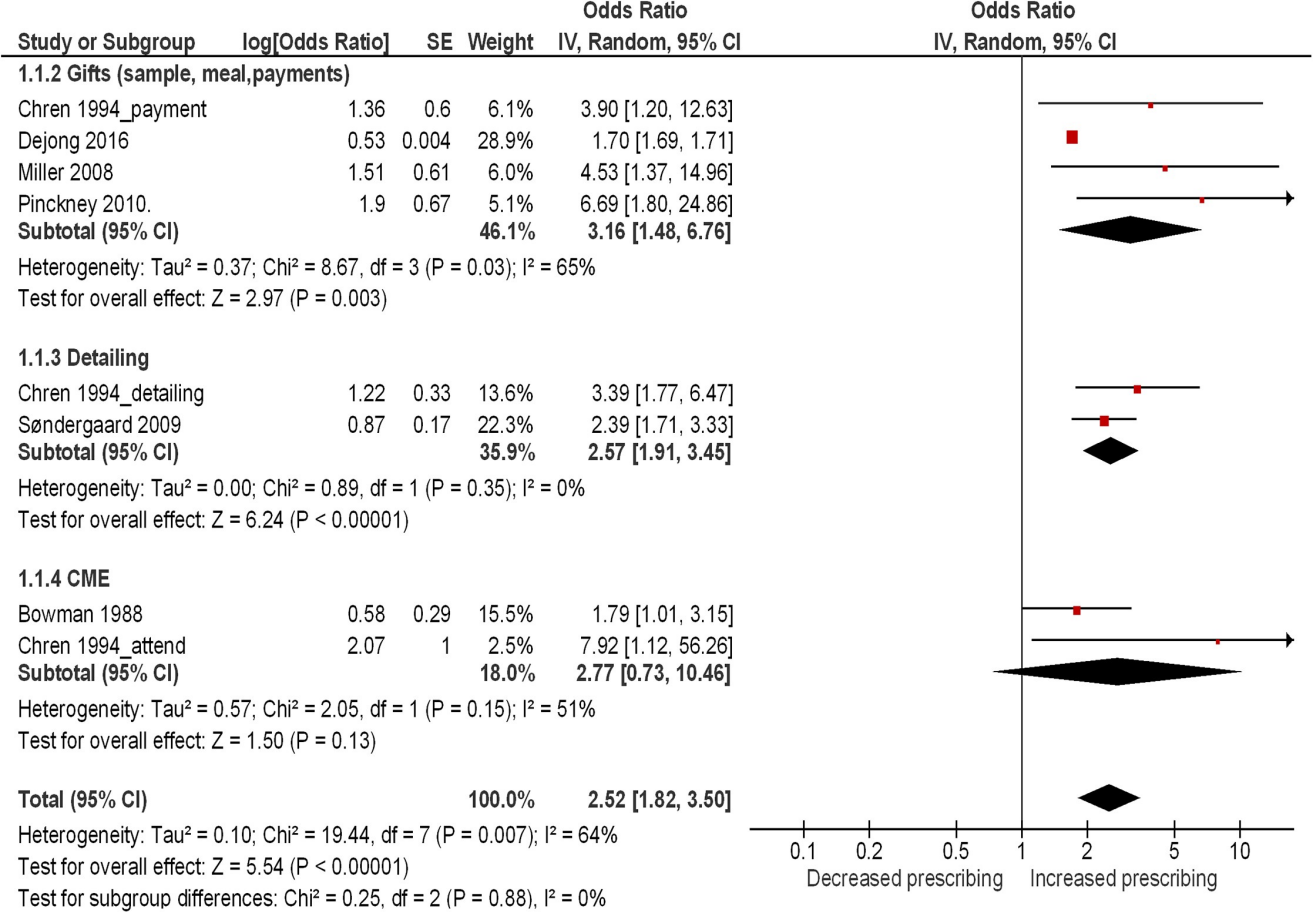
Forest plot for changes in physician prescribing behavior stratified by type of exposure.

We pooled the results for the six studies in a meta-analysis, stratified by type of exposure. Please refer to S3 Table for a summary of all decisions and their rationale with respect to the statistical data included in the meta-analysis. The pooled estimate showed a statistically significant association between interaction with pharmaceutical industry and physicians’ prescribing behaviors (OR = 2.52; 95% CI: 1.82–3.50). The heterogeneity was considered high with I^2^ of 64% (Fig 3). The test for subgroup effect did not identify any subgroup difference by type of exposure (Test for subgroup differences: P = 0.88).

**Fig 4:**
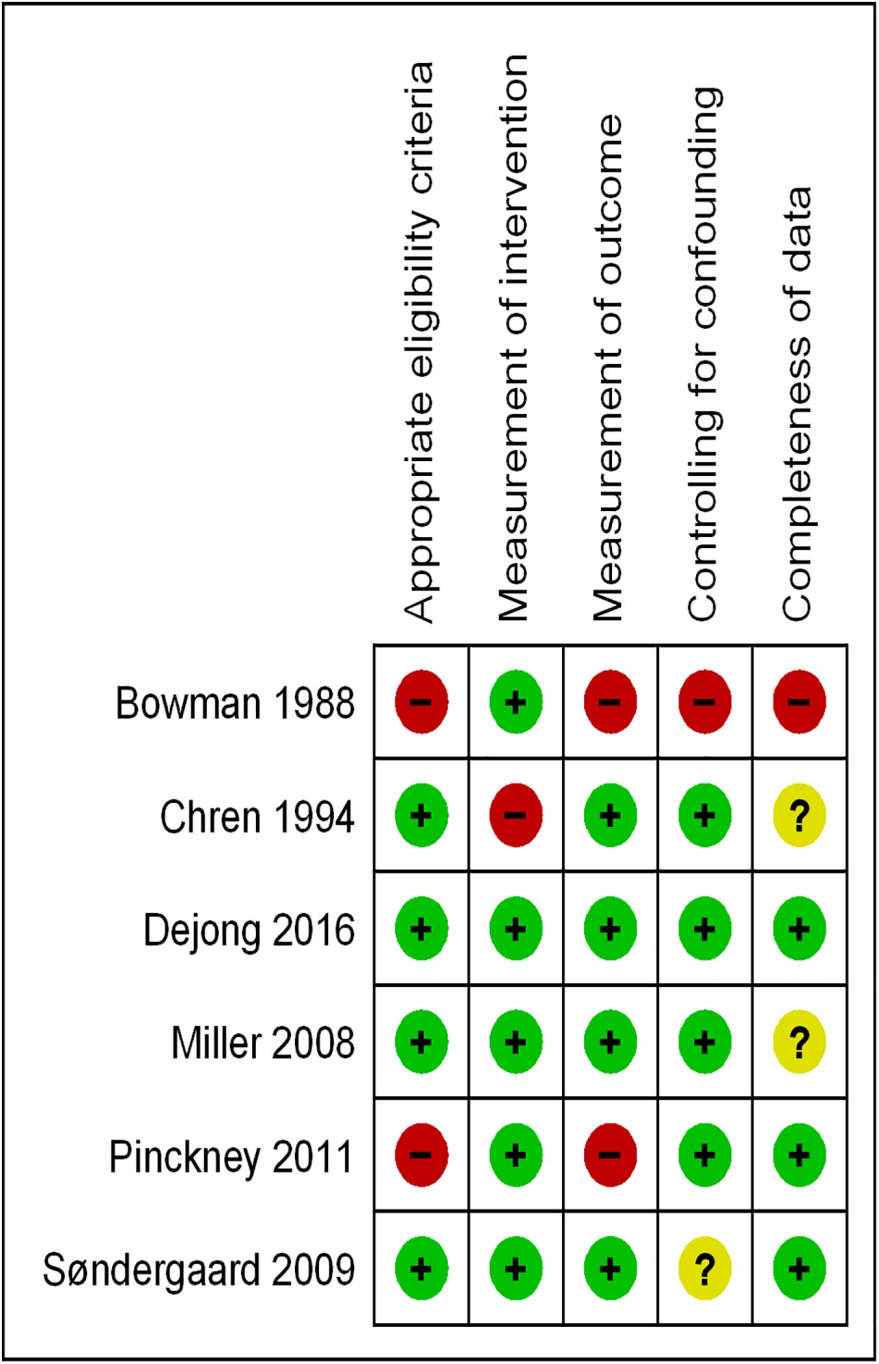
Risk of bias summary reflecting reviewers’ judgments about each risk of bias item for studies included in the meta-analysis.

Fig 4 shows the risk of bias summary for these six studies. We judged the overall risk of bias in four of these studies as low [15, 17, 28, 30]. Following the GRADE methodology we downgraded the quality of evidence for the outcome ‘behavior of physician’ from high to moderate for risk of bias and inconsistency. There was no major concern with imprecision, indirectness of the evidence, or publication bias warranting further downgrading.

The first sensitivity analysis excluded two studies judged as having an overall high risk of bias [19, 27]. The analysis found no important change in the pooled effect estimate (OR = 2.55, 95% CI: 1.75–3.71). In the second sensitivity analysis, we excluded the one study funded by pharmaceutical industry [17]. The analysis resulted in a non-substantial increase in size of the pooled effect estimate (OR = 2.74, 95% CI: 1.78–4.23). The third sensitivity analysis excluded two studies measuring the outcome of interest as ‘changes in generic prescription’ [28] and ‘formulary requests’ [30], respectively. The analysis resulted in the pooled effect estimate decreasing slightly in size but remaining statistically significant (OR = 2.11; 95% CI: 1.62–2.74). The details of the analyses are provided in S1, S2 and S3 Figs, respectively.”

### Narrative summary of studies not included in the meta-analysis

As noted above, fourteen eligible articles reporting thirteen studies were not included in the meta-analysis [12, 14, 16, 25, 26, 29, 31–37]. In addition, we narratively summarized the findings of the study by Pinckney et al (included in the meta-analysis), albeit only for the outcome ‘beliefs of physicians’ [19].

The study designs were cross-sectional (n = 12), pre-post survey (n = 1) [27] and retrospective cohort study (n = 1)[12]. These studies assessed the following types of interactions between physicians and drug representatives (with some studies reporting on more than one type): detailing (n = 8); continuing medical education including travel funding (n = 2); receiving free gifts (e.g. drug samples, meals, grants and payments) (n = 9); and a mix of interactions (n = 1; this study did not report data specific to each exposure). We summarized the results narratively, stratified by type of exposure and within each exposure by risk of bias.

#### Detailing (or pharmaceutical representative visits)

Nine articles reporting on eight studies evaluated the interactions between physicians and pharmaceutical sales representatives [12, 14, 25, 26, 34–37]. All of these studies assessed prescribing behaviors. In addition to assessing prescribing behavior, one assessed financial outcome [25] and one assessed physicians’ knowledge [37]. Five of the eight studies found an association between detailing and increased prescribing frequency, lower prescribing quality, higher expenditure, or earlier awareness of promoted drugs. Three studies found both associations with higher prescribing frequency or expenditures and lack of significant associations, for the different types of drugs or exposures examined. The overall risk of bias was judged as high for two of the eight studies [14, 37].

Figueiras et al. and Caamano et al. conducted a cross-sectional study to examine the association between detailing and the amount, expenditure and quality of drug prescribed by primary care physicians. The quality of prescription was reflected via three indicators which were combined to produce a global indicator variable. Figueiras et al. showed that using information obtained from pharmaceutical representatives was associated with a higher percentage of prescription drug not included in the primary care formulary and with a higher “global indicator variable”, thus reflecting lower prescription quality [26]. Camaano, et al., found that the utilization of the visiting marketers’ information was significantly associated with higher amounts of prescription (p = 0.048) and higher expenditure per physician (p = 0.035) [25]. On the contrary, the number of sales representative visits was not statistically associated with prescription amount or expenditure.

Pedan et al. conducted a retrospective cohort study to assess the impact of detailing on new prescriptions for three different statin brands, namely, Lipitor, Crestor and Vytorin [12]. The authors provided data for promotional activities of ‘own brand’ and the promotion of ‘competitive brand’. The findings for ‘own promotion’ indicated that detailing produced a highly significant positive impact on new prescriptions for Lipitor and Crestor (p<0.05), while results were not significant for Vytorin. ‘Competitive’ detailing had a significant negative impact on new prescription for Vytorin (p<0.05) whereas results were not significant for the other two brands.

Muijrers et al. conducted a cross-sectional study and found that more frequent visits from pharmaceutical industry representatives had a significant negative correlation with the quality of prescribing of general practitioners measured as “adherence to guidelines” (p<0.05) [36]. The latter was calculated as a weighted average score on 20 prescribing indicators based on general practice guidelines of the Dutch College of General Practitioners.

Mizik et al. conducted a pooled time series cross-sectional study to evaluate the association between pharmaceutical sales representative visits per month and the number of new prescriptions issued by a physician per month for three studied drugs [35]. The average number of new prescription per month by drug, were: 1.56 (95% CI: 0.80–2.23) for drug A; 0.32 (95% CI: 0.22–0.43) for drug B; and 0.15 (95% CI: 0.11–0.20) for drug C.

Becker et al. conducted a cross-sectional study and found that the use of detail men as sources of prescribing information concerning new drugs was significantly associated with higher prescription of the drug chloramphenicol by primary care physicians (p<0.01), and a poorer rating of prescription quality (p<0.01) by two panels of experts relative to five common complaints and five common illnesses [34].

The findings of the remaining two studies with overall high risk of bias are discussed below [14, 37].

Peay et al. conducted a cross-sectional study and found that doctors (specialists and general practitioners) who had contact with detail men regarding the promoted drug Temazepam reported earlier awareness of it (p < 0.041), were more likely to rate it as a moderate advance (as opposed to a minor advance or no advance at all) (p< 0.028), were more likely to have prescribed it (p< 0.0031), reported prescribing it earlier (p< 0.005) and were more likely to prescribe it routinely in preference to alternatives (p< 0.014) [37]. In the multivariate analysis, contact with detail man regarding Temazepam and first news of drugs from detail man remained significantly related to four of the five dependent variables correlating with prescription of Temazepam.

In another cross-sectional study, Lieb et al. found that frequently visited practices (i.e. daily or 2–3 times per week) had a significantly higher number of prescriptions (p = 0.005) and total daily doses per patient (p = 0.003) compared to practices visited less frequently by representatives. However, they did not have a higher total expenditure per patient (p = 0.115). The effects of the “frequency” of pharmaceutical sales representatives visits on prescribing behavior were no longer significant after taking into consideration the number of patients per office [14].

#### Continuing medical education

Two studies assessed the effects of industry-funded continuing medical education on physician prescribing behaviors [14, 29] and expenditure on off-patent branded drugs [14]. Both studies found significant associations between attending industry-funded continuing medical education and higher prescribing frequency, lower prescribing quality, or increased prescription cost. The overall risk of bias was judged as high for one of them [14].

A pre-post study tracked the prescribing pattern for two drugs before and after physicians attended symposia sponsored by a pharmaceutical company. The ‘expense-paid seminar at a resort’ was associated with a significant increase in the prescribing of the two promoted drugs within a few months of each symposium compared to their use before the symposium (p<0.001) [29].

The cross-sectional study with high risk of bias found that compared to doctors who frequently, occasionally or rarely took part in sponsored CME events, doctors who mentioned that they never took part in such events had a lower number of on patent-branded drug prescriptions per patient (mean ± SD; 1.05±0.35 vs. 1.27±0.55; p = 0.005, a higher proportion of generics (83.28±7.77% vs. 76.34±13.58%; p<0.0005) and lower expenditure on off-patent branded drugs per patient (£27.36±23.23 vs. £43.75±43.22; p = 0.002) [14].

#### Receiving free gifts

Nine studies evaluated receiving free gifts as the exposure of interest [12, 14, 16, 18, 19, 32, 33, 35, 37]. All of these studies assessed prescribing behaviors. In addition to assessing prescribing behaviors, two assessed financial outcomes [18, 32] and one assessed physicians’ beliefs [19]. For the latter study, we did not include the findings pertaining to prescribing behavior as these were already included in the meta-analysis. Five of the nine studies found an association between receiving free gifts and increased prescribing frequency, lower prescribing quality, or increased prescription cost. Each of three studies found both associations with higher prescribing frequency and lack of significant associations, for the different types of gifts examined in the studies. The remaining study found that prescribers with access to samples were significantly more likely to believe that samples benefited patients. The overall risk of bias was judged as high for four of the nine studies [14, 19, 33, 37].

Pedan et al. conducted a retrospective cohort study to evaluate the effects of sample dispensing and meals on total monthly number of new prescriptions written by a physician for the three leading statin brands (Crestor, Lipitor and Vytorin) [12]. The authors provided data for promotional activities of ‘own brand’ as well as the promotion of ‘competitive brand’. The findings for ‘own promotion’ showed that sample dispensing had a significant positive effect on new prescription for Crestor (p<0.01) and Vytorin (p<0.05), but results were not significant for Lipitor. They also found that free meals had a significant positive impact on new prescription for all three statin brands: Lipitor (p<0.05), Crestor (p<0.05) and Vytorin (p<0.01). ‘Competitive’ sampling had a significant negative impact on new prescription for Lipitor (p<0.05) and Vytorin (p<0.05) but results were not significant for Crestor. ‘Competitive’ free meal-related promotions had a significant negative impact on Lipitor only (p<0.05) The authors concluded that, while on average the marketing efforts affect the brand share positively, the magnitude of the effects is very brand specific.

Mizik et al. conducted a pooled time series cross-sectional study to examine the association between receiving free sample medications and the number of new prescriptions issued by a physician per month for three drugs [35]. They observed statistically significant but small effects of sample dispensing on prescription behavior for all three types of drugs.

In a cross-sectional study, Symm et al. examined the prescription claims data for 25 medications in one “clinic X” where sample medications were dispensed compared to two clinics, Y and Z, which did not dispense free sample medications [32]. They showed that, first, family physicians in clinic X significantly wrote the largest proportion of prescriptions for study medications (p <0.0001) versus non-study medications. Second, family physicians in clinic X significantly prescribed the lowest proportion of preferred name brands among study medications (p <0.0001). Third, the average cost of a 30-day prescription differed significantly by clinic (p <0.0001), being the highest in clinic X.

Hurley et al. conducted a cross-sectional study and found a strong correlation (r = 0.92) between the increase in provision of samples with a prescription by dermatologists and increased use of branded generic drugs promoted by these samples [18]. For physicians at local academic centers where free samples are prohibited, only 17% (230 of 1364) of the commonly prescribed medications were for branded or branded generic drugs compared to 79% for office-based dermatologists on a national level where free samples are available. Additionally, the national mean total retail cost of prescriptions was “conservatively” estimated to be twice as higher (roughly $465 nationally versus $200 at an academic medical center where samples were prohibited).

Yeh et al. found that among physicians with industry payments in the Massachusetts database, every $1000 in total payments received was associated with a 0.1% increase in the rate of brand-name statin drug prescribing (95% CI: 0.06%-0.13%; P < .001) [16]. Receiving payment for educational training was associated with an average 4.8% increase in prescribing of brand-name drugs (95% CI: 1.55–7.95; p = 0.004), but the other types of payment (i.e. food, grants/education gifts, and bona fide services) were not.

The findings of the four studies with overall high risk of biases are summarized below.

Peay et al. examined in a cross-sectional study the association between receiving a sample of Temazepam and physicians’ prescription rate and preference for it over the alternatives [37]. They demonstrated that physicians (specialists and general practitioners) who had received a sample of Temazepam, compared to those who had not, were more likely to have prescribed it (p < 0.001) and more likely to say that they now usually prescribe it rather than the alternatives (p < 0.006).

Pinckney et al. examined the association between the presence of samples in offices and prescribers’ beliefs about the use of samples. They found that prescribers with samples were significantly more likely to believe that samples: are liked by patients; expedite treatment; help patients who cannot afford their medication; reduce patient costs; and help physicians assess the efficacy of medications. Nonetheless, most prescribers with samples still agreed that samples alter treatment plans and increase the costs of care [19].

In another cross-sectional study, Lieb et al. found that physicians who always or frequently accepted gifts in the form of office stationery prescribed higher daily dose totals per patient (mean ± SD; 491.97±158.95 vs. 420.53±140.57; p = 0.003) and more generics (mean ±SD; 385.52±147.52 vs. 319.43±133.69; p = 0.004) in comparison to physicians who only occasionally, rarely or never accepted stationery [14]. We did not include the other types of gifts as the categorization of answer options was not conducive to interpretation (e.g., the categories ‘rarely’ ‘frequently’ and ‘occasionally’ were grouped together as exposure group and the category ‘never’ as control group for one type of gift whereas for another type of gift, the categories ‘frequently’ ‘occasionally’ ‘rarely’ and ‘never’ were categorized together as control group).

Anderson et al. conducted a cross-sectional study and found that the frequency of eating industry-funded food was associated with greater reliance of obstetrics and gynecology physicians on pharmaceutical representatives for drug information when prescribing new medications [33]. This finding was statistically significant in a first regression analysis (β = 0.16, 95% CI: 0.02–0.31). It became non-significant in a second regression model including as an independent variable “the perceived value of pharmaceutical representatives in helping physicians to learn about new drugs” (β = 0.07, 95% CI: -0.06–0.19).

#### Mixed exposures

One cross-sectional study with overall high risk of bias examined the association between rational prescribing of general practitioners and a mix of exposures including “manufacturer’s representatives, drug companies’ mailings, use of samples, drug companies’ journals, drug firm meetings and usefulness of drug company information” [31]. The investigators found that reliance on information from pharmaceutical industry was negatively associated with prescribing rationality (p<0.001).

## Discussion

### Summary and interpretation of findings

We identified 19 studies looking the association between physicians’ interactions with pharmaceutical companies and their prescribing behaviors. In addition to assessing prescribing behavior, four of the 19 studies assessed financial outcomes [12, 19, 26, 35], one assessed physicians’ knowledge [37], and one assessed their beliefs [19]. None of the included studies assessed clinical outcomes.

Out of the 19 studies, 15 found a consistent association between interactions promoting a medication, and inappropriately increased prescribing rates, lower prescribing quality, and/or increased prescription costs. Each of three studies found both associations with higher prescribing frequency and lack of significant associations, for the different types of exposures and drugs examined in the studies [12, 16, 25]. Only one study, albeit at high risk of bias, found an association between receiving gifts from the industry and increased generic prescribing whereas the results were mixed for the remaining types of exposures and outcomes assessed in that study [14].

A meta-analysis of six of the included studies provided moderate quality evidence showing more than doubling of inappropriate prescribing rate among practicing physicians. The heterogeneity was considered high. Sensitivity analyses excluding the two studies at high risk of bias and the industry-funded study, respectively, did not substantially change these results. A subgroup analysis did not find a difference by type of exposure.

### Strengths and limitations of the review

A major strength of the present study is the use of Cochrane methodology for conducting the systematic review. In addition, this is the first systematic review to focus on practicing physicians, as other reviews included residents and physicians in training as their population of interest [11]. A potential limitation of our review is that we searched only two electronic databases. However, we believe our search was sensitive. Also, Spurling et al. did not include a study that was eligible for our review, and that was missed by our search strategy. Another potential limitation is the exclusion of studies published in a language other than English. Also, all included studies were conducted in developed, high-income countries; therefore, there is a chance that we have missed studies conducted in low or middle-income countries that have been published in a non-English language. However, given the consistency of findings, we expect them to be generalizable to those countries. Another limitation relates to the potential misattribution of the exposure category; however, such cases were few. One example is the study conducted by Yeh et al. which considered “payments for CME” as different from “payments for educational training” [16]. Other limitations relate to the observational nature of all included studies and to our inability to include some studies in the meta-analysis because they did not report data for the association between the exposure and the outcome of interest.

### Comparison to findings of similar reviews

The latest systematic review addressing the same topic was published in 2010 and included physicians as well as residents [11]. The chosen populations may have contributed to the high level of statistical heterogeneity reported (I^2^ = 91%). Also, the previous systematic review focused on exposure to information directly provided by pharmaceutical companies, and thus excluded other types of exposures such as gifts, samples, and continuing medical education courses that were funded by unrestricted grants from pharmaceutical companies. On the other hand, our review has included these types of interaction and has covered 8 years of literature since 2008, the year of literature search of the 2010 review. Still, our review included a relatively lower number of studies compared to the previous review due to the stricter eligibility criteria (e.g., we excluded residents and medical trainees; non-targeted interactions such as advertisements in journals or prescribing software, or mailed information; and participation in sponsored clinical trials). Nevertheless, our findings are consistent with those of the previous review in terms of decreased prescribing quality and increased costs associated with exposure to information provided directly by pharmaceutical companies. In addition, we found evidence of similar effects associated with drug samples and industry gifts (both of which were not assessed in the previous review).

### Implications for policy and practice

Our findings suggest that the interaction between physicians and pharmaceutical companies should be better managed to reduce any negative effects and promote appropriate drug prescribing. While many policy options have been proposed to manage the interactions between physicians and pharmaceutical companies [38], the level of evidence supporting them varies, as described below.

There is an increasing trend of mandating pharmaceutical companies to disclose payments to physicians. For instance, the Physician Payments Sunshine Act enacted in the USA in 2010 requires pharmaceutical and medical device manufacturers to publicly disclose payments (or transfers of values) exceeding US$10 per instance or US$100 per year made to physicians and teaching hospitals [39]. Similarly, Medicines Australia recently revised its code of conduct, requiring pharmaceutical companies to report on payments to individual health professionals for their services, sponsorships to attend educational events, as well as educational grants [40]. However, so far there is no evidence supporting the effectiveness of mandatory disclosure [20].

As an example of regulatory approach, France introduced in 2004 the French Sales Visit Charter which requires sales representatives to provide physicians with “approved product information” [41]. A report by French National Authority for Health pointed to the ineffectiveness of the Charter and the difficulty in supervising the content of verbal information conveyed during representative visits [42].

A potentially effective option would be to restrict physician-industry interactions, particularly given the evidence that restriction policies may have a positive effect on improving prescribing behavior [20]. This could be achieved, at the institutional level by restricting free samples, promotional material, and meetings with pharmaceutical company representatives. For example, Stanford University banned pharmaceutical sales representatives from its hospitals [43]. Similarly, Memorial Sloan Kettering Cancer Center and Brody School of Medicine at East Carolina University banned all industry involvement (including funding) in CME, reportedly with success [44]. At a higher level, policy makers may consider legislation specifying the types of interactions that are permissible, and those that are not. For example, Minnesota enacted a law that bans pharmaceutical industries from providing gifts to physicians with a total annual combined retail value above US$50 [38].

While some may claim that restricting interactions between physicians and pharmaceutical companies could create an ‘information gap’, several studies conducted in different contexts found that sales representatives often did not state the risks and harmful effects of drugs to physicians [40, 41, 45]. Academic detailing has emerged as an effective alternative to industry-dependent drug information [46, 47]. For instance, Canada and some USA states have established nationally-funded academic detailing programs that rely on similar sales tactics utilized by the pharmaceutical industry to influence physician prescribing according to evidence-based guidelines [48].

Considerations should also be given to educating health care providers about the influence of interactions with the pharmaceutical industry, as well as inclusion of courses on industry marketing techniques and conflict of interest in medical curricula [49]. Existing evidence suggests positive effects of educational programs about industry marketing strategies on medical trainees’ attitudes and behaviors [50, 51].

### Implications for research

Given all of the included studies were conducted in high-income countries, future studies should explore the effect of interaction of physicians in low and middle-income countries with pharmaceutical companies on their clinical practices. Also, it would be important to understand the impact of these interactions on clinical outcomes.

## Supporting information

S1 Appendix Study protocol. (PDF)

S2 Appendix PRISMA Checklist. (DOC)

S3 Appendix Full search strategy. (PDF)

S1 Table A summary of author contacts. (PDF)

S2 Table A list of the excluded studies along with reasons for exclusion. (PDF)

S3 Table A summary of all decisions and their rationale with respect to the statistical data included in the meta-analysis. (PDF)

S1 Fig Sensitivity analysis- exclusion of high risk of bias studies. (TIF)

S2 Fig Sensitivity analysis-exclusion of industry-sponsored study. (TIF)

S3 Fig Sensitivity analysis- exclusion of studies with indirect measures of outcomes. (TIF)
