# Supplementary material for: Association between physicians’ interaction with pharmaceutical companies and their clinical practices: A systematic review and meta-analysis
Source: PLoS One. 2017 Apr 13;12(4):e0175493. doi: 10.1371/journal.pone.0175493 (PMC5391068; doi:10.1371/journal.pone.0175493)
Supplement: S1 Appendix — (PDF) [file pone.0175493.s001.pdf]

Association between physicians' interaction with pharmaceutical companies and their clinical practices: a systematic review protocol

Hneine Brax, Racha Fadlallah, Lina al-Khaled, Lara Kahale, Hala Nas, Fadi El-Jardali, Elie A. Akl,

Review question(s)
Pharmaceutical company representatives likely influence the prescribing habits and professional behaviors of physicians. The objective of this study was to systematically review the evidence on the association between physicians' interactions with pharmaceutical companies and their clinical practices.

Searches
Search of electronic databases, July 2016: MEDLINE, EMBASE, Screening of the reference lists of included studies. Contact of experts: we attempt to contact the authors of relevant articles when needed. We exclude non English studies.
We will not exclude studies based on date of publication.

Types of study to be included
Types of study designs: observational (e.g., cohort, time series analysis, before-after design, case control, cross sectional), and experimental (non-randomized controlled trials, and randomized controlled trials).

We will exclude ecological studies, econometric studies, editorials, letters to the editor, and non-English studies

Participants/ population
Practicing physicians (as defined in the primary studies)

We will exclude studies focusing on medical students and physicians in training.

Exposure(s)
Active interaction between physicians and drug companies (e.g., detailing; industry-sponsored continuous medical education; receiving free drug samples, industry provided meals, gifts, pens)

We will exclude studies assessing passive interactions such as journal advertisement.
We will also exclude studies assessing industry-independent drug information interventions or interventions to reduce interactions between physicians and pharmaceutical companies.

Comparator(s)/ control
Either no interaction or a lower level of interaction.

Context
Setting: high, low and middle income countries. No restriction.

Outcome(s)
- Physicians' knowledge;
- Physicians' attitude;
- Physicians' behavior;
- Financial outcomes;
- Patients' clinical outcomes.

Data extraction, (selection and coding)

Selection process:

Title and abstract screening: Teams of two reviewers will use the above eligibility criteria to screen titles and abstracts of identified citations in duplicate and independently for potential eligibility. We will get the full text for citations judged as potentially eligible by at least one of the two reviewers.

Full-text screening: Teams of two reviewers will use the above eligibility criteria to screen the full texts in duplicate and independently for eligibility. The teams of two reviewers will resolve disagreement by discussion or with the help of a third reviewer.

We will use standardized and pilot tested screening forms. We will conduct calibration exercises to ensure the validity of the selection process.

<u>Data abstraction process:</u>
Teams of two reviewers will abstract data from eligible studies in duplicate and independently. They will resolve disagreements by discussion or with the help of a third reviewer.

We will collect the following data: type of study, funding source, characteristics of the population, exposure, outcomes assessed, and statistical data.

We will use standardized and pilot tested data abstraction forms
We will conduct calibration exercises to ensure the validity of the data abstraction process

Risk of bias (quality) assessment

Teams of two reviewers will assess the risk of bias in each study in duplicate and independently. They will resolve disagreements by discussion or with the help of a third reviewer.

We will use the Cochrane Risk of Bias tool to assess the risk of bias in randomized trials.

We will use the tool suggested by the GRADE working group for assessing the risk of bias for observational studies [16]. We will calculate the risk of bias using the following criteria:

- Failure to develop and apply appropriate eligibility criteria (e.g., no clear eligibility criteria, convenient sampling, under- or over-matching in case-control studies, selection of exposed and unexposed in cohort studies from different populations, and low response rate).
- Flawed measurement of exposure (e.g., differences in measurement of exposure such as recall bias in case- control studies, , and subjective or self-reported assessment of exposure)
- Flawed measurement of outcome (e.g., differential surveillance for outcome in exposed and unexposed in cohort studies, and subjective or self-reported assessment of outcome)
- Failure to adequately control confounding (e.g., failure of accurate measurement of all known prognostic factors, failure to match for prognostic factors and/or adjustment in statistical analysis
- Incomplete follow-up or failure to control for loss-to-follow up

We will grade each potential source of bias as high, low or unclear risk of bias. We will use unclear when the authors did not report enough information for us to make the judgment.

We will not exclude any study based on quality. Instead, we will downgrade the quality of evidence using GRADE approach.

Strategy for data synthesis

We will calculate the agreement between reviewers for the assessment of study eligibility (at the full text screening stage) using kappa statistic.

We will conduct a meta-analysis to pool the results across studies for the association between ‘targeted interactions with physicians’ as the exposure of interest, and ‘changes in physician prescribing behavior’ as the outcome of interest.

We will use the following a priori plan for choosing which data to include in the meta-analysis:

- For studies reporting on more than one type of exposure (e.g., gifts, detailing), we treated each exposure as a separate unit of analysis.
- For studies measuring the same outcome at several points in time, we chose the first time point to avoid any potential confounding effects from subsequent measures.

- For studies assessing the association of interest for more than one drug (i.e., reporting more than one association), we included the value that is the closest to the mean of all reported values amongst those associations.

We will use the generic inverse variance technique with a random-effects model to pool the association measures across included studies that reported the needed statistical data. We will carry out statistical analysis using RevMan (version 5.2). For categorical data, we will calculate the ORs for each study. For continuous data, we will calculate the mean difference (or, when appropriate, the standardized mean difference) for each study. We will consider changes in physician prescribing behavior as the outcome measure and active interactions with physicians as the exposure of interest.

To take into account the heterogeneity introduced by the different types of exposures (i.e., gifts, detailing, and CME), we will stratify the meta-analyses by type of exposure. We will test the results for homogeneity using the I2 test and considered heterogeneity present if I2 exceeded 50%.

We will create inverted funnel plots of individual study results plotted against sample size in order to check for possible publication bias. We will also report the results narratively and stratified by type of intervention

We will report the results of studies that we could not include in the meta-analysis in a narrative way.

We will then assess the quality of evidence using the GRADE approach.

Dissemination plans
We will publish results in international, peer-reviewed journals.
