# Supplementary material for: Association between physicians’ interaction with pharmaceutical companies and their clinical practices: A systematic review and meta-analysis
Source: PLoS One. 2017 Apr 13;12(4):e0175493. doi: 10.1371/journal.pone.0175493 (PMC5391068; doi:10.1371/journal.pone.0175493)
Supplement: S3 Appendix — (PDF) [file pone.0175493.s003.pdf]

S2 Appendix: full search strategy

Database: Ovid MEDLINE(R) <1946 to July Week 3 2016>
Search Strategy:
----
1 Conflict of Interest.mp. or "Conflict of Interest"/ (9575)

2 Drug Industry/ (30180)

3 Gift Giving/ (1385)

4 detailman.mp. (4)

5 commercial information.mp. (33)

6 ((drug or pharma*) adj3 (industry or firm* or manufacture* or compan*)).mp. (41596)

7 physician*.mp. (443378)

8 doctor*.mp. (93268)

9 Physicians/ (76762)

10 primary care.mp. (77107)

11 or/1-6 (49715)

12 or/7-10 (545360)

13 11 and 12 (5810)

14 13 not (comment or editorial or letter).pt. (4869)

****

Database: Embase <1980 to 2016 Week 31>
Search Strategy:
----
1 Conflict of Interest.mp. or "Conflict of Interest"/ (10662)

2 Drug Industry/ (71418)

3 Gift Giving/ (1003)

4 detailman.mp. (3)

5 commercial information.mp. (50)

6 ((drug or pharma*) adj3 (industry or firm* or manufacture* or compan*)).mp. (102994)

7 physician*.mp. (556336)

8 doctor*.mp. (211287)

9 Physician/ (228048)

10 primary care.mp. (111197)

11 or/1-6 (112316)

12 or/7-10 (755418)

13 11 and 12 (8715)

14 13 not (comment or editorial or letter).pt. (7531)

****
