# Supplementary material for: Association between physicians’ interaction with pharmaceutical companies and their clinical practices: A systematic review and meta-analysis
Source: PLoS One. 2017 Apr 13;12(4):e0175493. doi: 10.1371/journal.pone.0175493 (PMC5391068; doi:10.1371/journal.pone.0175493)
Supplement: S1 Table — (PDF) [file pone.0175493.s004.pdf]

S1 Table: Summary of contacting authors of included studies for additional information or data

Study Name	Information requested	Response
Becker, 1972 [1]	Odds ratio or raw data to calculate odds ratio for the association between use of detail men as sources of prescribing information and prescription of chloramphenicol	Two reminders were sent but author didn't reply back
Peah 1988 [2]	Odds ratio or raw data to calculate odds ratio for the association between detailmen and prescription patter, and sample size and prescription pattern	We couldn't find the emails of any of the authors even after a detailed search
Orlowski 2002 [3]	Whether values reported are for standard deviation or standard error	Author confirmed that standard deviation was used in the analysis
Caamano 2002 [4]	Odds ratio for the association between sales visit and prescription amount	Author reported he was not able to find requested data
Mizik 2005[5]	Data to calculate the Odds ratio for association between sampling and new prescription, and detailing and new prescription, for drugs A, B, C respectively	Author is unable to share data because she does not have the permission of the data owner to do so.
Muijres, 2005 [6]	Data to calculate means and SD	Author stated that he has retired since 2008
Symm , 2006 [7]	sample medication were dispensed) significantly wrote the largest proportion of prescriptions for study medications	Author reported that he can't provide the data
Pedan 2011[8]	Whether values reported are for standard deviation or standard error	Author clarified that standard errors were used in the analysis
Hurley, 2014 [9]	N values to calculate the mean and SD for office-based dermatologists on a national level where free samples are available.	Author did not reply back

References

1. Becker MH, Stolley PD, Lasagna L, McEvilla JD, Sloane LM: **Correlates of physicians' prescribing behavior**. *Inquiry* 1972, **9**(3):30-42.
2. Peay MY, Peay ER: **The role of commercial sources in the adoption of a new drug**. *Social science & medicine* 1988, **26**(12):1183-1189.
3. Orlowski JP, Wateska L: **The effects of pharmaceutical firm enticements on physician prescribing patterns. There's no such thing as a free lunch**. *Chest* 1992, **102**(1):270-273.
4. Caamano F, Figueiras A, Gestal-Otero JJ: **Influence of commercial information on prescription quantity in primary care**. *Eur J Public Health* 2002, **12**(3):187-191.
5. Mizik N JR: **Are Physicians "Easy Marks"? Quantifying the Effects of Detailing and Sampling on New Prescriptions.** *Management Science* 2004, **50**(12):1704-1715.
6. Muijrers PE, Grol RP, Sijbrandij J, Janknegt R, Knottnerus JA: **Differences in prescribing between GPs: impact of the cooperation with pharmacists and impact of visits from pharmaceutical industry representatives**. *Fam Pract* 2005, **22**(6):624-630.
7. Symm B AM, Forjuob SN, Preece: **Effects of using free sample medications on the prescribing practices of family physicians**. *J Am Board Fam Med* 2006, **19**:443–449.
8. Pedan A, Wu H: **Asymmetric responsiveness of physician prescription behavior to drug promotion of competitive brands within an established therapeutic drug class**. *Health Mark Q* 2011, **28**(2):133-154.
9. Hurley MP, Stafford RS, Lane AT: **Characterizing the relationship between free drug samples and prescription patterns for acne vulgaris and rosacea**. *JAMA dermatology* 2014, **150**(5):487-493.
