# Supplementary material for: Association between physicians’ interaction with pharmaceutical companies and their clinical practices: A systematic review and meta-analysis
Source: PLoS One. 2017 Apr 13;12(4):e0175493. doi: 10.1371/journal.pone.0175493 (PMC5391068; doi:10.1371/journal.pone.0175493)
Supplement: S2 Table — (PDF) [file pone.0175493.s005.pdf]

S2 Table: List of excluded studies along with reasons for exclusion

Full-text articles excluded, with reasons (n=85)

Qualitative study (n= 6)
Ecological study (n= 7)
Not a primary study (n= 11)
No association of interest (n=6)
No control group of interest (n= 8)
No exposure of interest (n= 12)
No outcome of interest (n= 8)
Not industry related (n= 3)
Measures attitude towards physician-industry interaction (n= 9)
Focuses on medical student/residents/trainees (n= 4)
Non-English study (n= 4)
Econometric study (n=1)
Intervention studies (n=6)

**Study**	**Reason for Exclusion**
Ahmadi 2015(1)	Not a primary study
Al-Areefi 2013(2)	No exposure of interest<br>(study examines whether relationship between perception of PRS by physician and their prescribing behaviors were associated)
Alsageer 2012(3)	No outcome of interest<br>(study examined the extent of doctors' involvements in PCRs' activities and the factors associated with such engagement, without linking such interactions to changes in knowledge, attitude or behavior of physicians)
Andaleeb 1996(4)	Measures attitude towards physician-industry interaction
Anderson 2015(5)	Intervention study
Anonymous 2015(6)	Intervention study
Babu 2012(7)	No exposure of interest
Backer 2000(8)	Qualitative study
Berings and Habraken, 1994(9)	Not industry related<br>(Industry-independent drug information interventions)
Bogenmann 2015 (10)	No exposure of interest (surrogate exposure)
Boltri, 2002 (11)	Intervention study
Burashnikova 2008(12)	Measures attitude towards physician-industry interaction

Caudill 1996(13)	No association of interest<br>(Study only measured the association between attitudes towards pharmaceutical representative and frequency of use of information provided by pharmaceutical representative)
Chew 2000(14)	Study focused on medical students
Chimonas 2007(15)	Qualitative study
Chressanthis 2014 (16)	Ecological study<br>(study looked at the association between organizational characteristics and actual adoption and not at the level of physician)
Christensen 1979(17)	No association of interest
Darmon 2015(18)	Non-English study
De Angelis(19)	Not a primary study
De Ferrari 2014(20)	No outcome of interest<br>(study did not relate exposure to outcome)
De la Cauvas 2002(21)	No control group of interest
Donohue 2004(22)	Ecological study<br>(measure of exposure was ecological)
Doval 2015(23)	Measures attitude towards physician-industry interaction
Fleischman 2016(24)	Ecological study
Ferguson 1999(25)	No outcome of interest
Freeman 1993(26)	No exposure of interest
Freemantle et al, 2000 (27)	Intervention study
Fretheim 2005(28)	Ecological study
Gonul 2001(29)	No control group of interest
Gönül 2010(30)	No control group of interest<br>(compared E-detailing to traditional detailing)
Gunnarsdottir 2005(31)	No exposure of interest
Halperin, 2004(32)	Measures attitude towards physician-industry interaction
Hansen 2010(33)	Ecological study<br>(study unable to capture person-level variation in promotional spending)
Hemminki 2004(34)	No exposure of interest
Henry 2002(35)	No outcome of interest
Herrera 2004(36)	Non-English study
Hartung 2010(37)	Intervention study

Hossain 2013(38)	No association of interest
Ichikawa 2016(39)	Not a primary study (commentary)
Iizuka 2007(40)	No exposure of interest
Indhumathi 2015(41)	No outcome of interest
Joyce 2011(42)	No outcome of interest (study focused more on whether broad or narrow prescribing is associated with patient-level outcomes)
Kamal 2015(43)	Measures attitude towards physician-industry interaction
Kannan 2015(44)	Measures attitude towards physician-industry interaction
Khajuria 2013(45)	No control group of interest<br>(study involved rating various pharmaceutical communication strategies by physicians using five point Likert scale ranging from “Not At All Important effective” (score =1) to “Very Important” (score =5))
Kinoshita 2015(46)	Focus on medical student/residents/trainees
Klemenc-Ketis 2013(47)	No exposure of interest
Knudsen 2014(48)	Ecological study<br>(study looked at the association between organizational characteristics and actual adoption and not at the level of physician)
Lee 2016(49)	No outcome of interest
Lenzen 2016(50)	Non-English study
Lenzer 2015(51)	Not a primary study
Lurk 2004(52)	No outcome of interest
Lussier 2015(53)	Non-English study
Mackowiak 1985(54)	Ecological study
Maeda 2015 (55)	Not industry-related
Mansfield 2005(56)	Not a primary study
Mansfield 2008(57)	Not a primary study
McMahon 2015(58)	Not a primary study
McGavock 1993(59)	No exposure of interest
Miao-Sheng 2008(60)	No exposure of interest<br>(study focused on sales discount as a marketing strategy)
Narendran 2013(61)	No control group of interest<br>(Study involved rating the effectiveness of different marketing strategies (on a scale of 1-7) by physicians and sales personnel)

Newton-Syms 1992(62)	Not industry-related<br>(industry-independent drug information interventions)
O'Dowd 2015(63)	Not a primary study
Peah, 1984(64)	Measures attitude towards physician-industry interaction
Prosser 2003(65)	Qualitative study
Prosser 2003(66)	Qualitative study
Rahman 2015(67)	Qualitative study
Ridley 2015(68)	No exposure of interest
Riese 2015(69)	Focuses on medical students/residents/trainees
Sarpatwari 2015(70)	No association of interest
Sharma 2016(71)	Not a primary study<br>(presented only as abstract)
Skipper 2015(72)	No association of interest
Spurling 2007(73)	Intervention study
Spurling 2010(74)	Not a primary study<br>(systematic review)
Stark 2016 (75)	Measures attitude towards physician-industry interaction
Steinman 2007(76)	No control group of interest
Stelfox 1998(77)	Focuses on medical students/residents/trainees<br>(results of interest not restricted to practicing physicians)
Stoddard 2003(78)	No association of interest
Tahmasebi 2015 (79)	Econometric study (no targeted interaction)
Turner 1986(80)	Not a primary study (narrative study)
Tahmasebi 2015(81)	No exposure of interest
Taylor 1991(82)	No control group of interest
Tengilimoglu 2005(83)	No association of interest
Tobin 2008(84)	Qualitative study
Wilson 1963(85)	No control group of interest

References of excluded studies

1. Ahmadi Nasab Emran S. Assessing Information from Pharmaceutical Company Representatives. AMA J Ethics. 2015;17(8):729-33.
2. Al-Areefi MA, Hassali MA, Mohamed Ibrahim MI. The role of pharmaceutical marketing and other factors in prescribing decisions: the Yemeni experience. Research in social & administrative pharmacy : RSAP. 2013;9(6):981-8.
3. Alssageer MA, Kowalski SR. A survey of pharmaceutical company representative interactions with doctors in Libya. The Libyan journal of medicine. 2012;7.
4. Anadaleeb SS, Tallman RF. Relationships of physicians with pharmaceutical sales representatives and pharmaceutical companies: an exploratory study. Health Mark Q. 1996;13(4):79-89.
5. Anderson TS, Huskamp HA, Epstein AJ, Barry CL, Men A, Berndt ER, et al. Antipsychotic prescribing: do conflict of interest policies make a difference? Med Care. 2015;53(4):338-45.
6. Anonymous. No-gift policy in medical schools. Demonstrated impact on prescribing behaviour. Prescrire Int. 2015;24(159):111.
7. Babu MA, Heary RF, Nahed BV. Device innovation in neurosurgery: controversy, learning, and future directions. Neurosurgery. 2012;70(4):789-94; discussion 94-5.
8. Backer EL, Lebsack JA, Van Tonder RJ, Crabtree BF. The value of pharmaceutical representative visits and medication samples in community-based family practices. J. 2000;49(9):811-6.
9. Berings D, Blondeel L, Habraken H. The effect of industry-independent drug information on the prescribing of benzodiazepines in general practice. Eur J Clin Pharmacol. 1994;46(6):501-5.
10. Bogenmann L, Vidal P, Pereira B, Tauveron I. Characteristics of practitioners prescribing glucagon-like peptide-1 receptor agonists and dipeptidyl peptidase-4 inhibitors. [French]. Medecine des Maladies Metaboliques. 2015;9(3):311-5.
11. Boltri, J. M., Gordon, E. R., & Vogel, R. L. (2002). Effect of antihypertensive samples on physician prescribing patterns. Family medicine, 34(10), 729-731.
12. Burashnikova IS, Ziganshin AU, Ziganshina LE. Attitudes to pharmaceutical promotion techniques among healthcare professionals in the Republic of Tatarstan, Russia. International Journal of Risk and Safety in Medicine. 2008;20(1-2):57-71.
13. Caudill TS, Johnson MS, Rich EC, McKinney WP. Physicians, pharmaceutical sales representatives, and the cost of prescribing. Arch Fam Med. 1996;5(4):201-6.
14. Chew LD, O'Young TS, Hazlet TK, Bradley KA, Maynard C, Lessler DS. A physician survey of the effect of drug sample availability on physicians' behavior. J Gen Intern Med. 2000;15(7):478-83.
15. Chimonas S, Brennan TA, Rothman DJ. Physicians and drug representatives: exploring the dynamics of the relationship. J Gen Intern Med. 2007;22(2):184-90.
16. Chressanthis, G.A., Sfekas, A., Khedkar, P., Jain, N. and Poddar, P., 2014. Determinants of pharmaceutical sales representative access limits to physicians. Journal of Medical Marketing, 14(4), pp.220-243.
17. Christensen DB, Wertheimer AI. Sources of information and influence on new drug prescribing among physicians in an HMO. Social science & medicine. 1979;13A(3):313-22.
18. Darmon D, Belhassen M, Quien S, Langlois C, Staccini P, Letrilliart L. [Factors associated with drug prescription in general practice: a multicenter cross-sectional study]. Sante Publique. 2015;27(3):353-62.
19. De Angelis CD. The Importance of Physicians' Financial Disclosure for the Public's Health. Milbank Quarterly. 2015;93(4):679-82.
20. De Ferrari A, Gentille C, Davalos L, Huayanay L, Malaga G. Attitudes and relationship between physicians and the pharmaceutical industry in a public general hospital in Lima, Peru. PloS one. 2014;9(6):e100114.
21. De Las Cuevas C, Sanz EJ, De La Fuente JA. Variations in antidepressant prescribing practice:

clinical need or market influences? Pharmacoepidemiol Drug Saf. 2002;11(6):515-22.
22. Donohue JM, Berndt ER, Rosenthal M, Epstein AM, Frank RG. Effects of pharmaceutical promotion on adherence to the treatment guidelines for depression. Med Care. 2004;42(12):1176-85.

23. Doval HC, Tajer CD, Borracci RA, Nunez C, Samarelli M, Tamini S. Survey on Ethical Conduct Thresholds in Cardiologal Medical Practice in Argentina. Developing World Bioeth. 2015;15(2):68-75.
24. Fleischman W, Agrawal S, King M, Venkatesh AK, Krumholz HM, McKee D, et al. Association between payments from manufacturers of pharmaceuticals to physicians and regional prescribing: cross sectional ecological study. Bmj. 2016;354:i4189.
25. Ferguson RP, Rhim E, Belizaire W, Egede L, Carter K, Lansdale T. Encounters with pharmaceutical sales representatives among practicing internists. Am J Med. 1999;107(2):149-52.
26. Freeman JK, Barnes JH, Summers KH, Szeinbach SL. Modeling physicians' prescribing decisions for patients with panic disorder. J Health Care Mark. 1993;13(1):34-9.
27. Freemantle N, Johnson R, Dennis J, et al. Sleeping with the enemy? A randomized controlled trial of a collaborative health authority/ industry intervention to influence prescribing practice. Br J Clin Pharmacol 2000;49:174–9
28. Fretheim A, Oxman AD. International variation in prescribing antihypertensive drugs: its extent and possible explanations. BMC Health Serv Res. 2005;5(1):21.
29. Gönül F, Carter, F., Petrova, E., Srinivasan, K. . Promotion of Prescription Drugs and Its Impact on Physicians' Choice Behavior. Journal of Marketing. 2001;65(3):79-90.
30. Gonul FF, Carter FJ. Impact of e-detailing on the number of new prescriptions. Health Care Manag Sci. 2010;13(2):101-11.
31. Gunnarsdottir AI, Kinnear M. Factors that influence prescribers in their selection and use of COX-2 selective inhibitors as opposed to non-selective NSAIDs. Pharm World Sci. 2005;27(4):316-20.
32. Halperin EC, Hutchison P, Barrier RC, Jr. A population-based study of the prevalence and influence of gifts to radiation oncologists from pharmaceutical companies and medical equipment manufacturers. Int J Radiat Oncol Biol Phys. 2004;59(5):1477-83.
33. Hansen RA, Chen SY, Gaynes BN, Maciejewski ML. Relationship of pharmaceutical promotion to antidepressant switching and adherence: a retrospective cohort study. Psychiatr Serv. 2010;61(12):1232-8.
34 Hemminki E, Karttunen T, Hovi SL, Karro H. The drug industry and medical practice--the case of menopausal hormone therapy in Estonia. Social science & medicine. 2004;58(1):89-97.
35. Henry D, Lexchin J. The pharmaceutical industry as a medicines provider. Lancet. 2002;360(9345):1590-5.
36. Galan Herrera S, Delgado Marroquin MT, Altisent Trota R. [Analysis of the relationship between primary care doctors and the pharmaceutical industry]. Atencion primaria / Sociedad Espanola de Medicina de Familia y Comunitaria. 2004;34(5):231-7.
37. Hartung, D. M., Evans, D., Haxby, D. G., Kraemer, D. F., Andeen, G., & Fagnan, L. J. (2010). Effect of drug sample removal on prescribing in a family practice clinic. The Annals of Family Medicine, 8(5), 402-409.
38. Hossain MK, S., Tanmy, T., Yousuf, A. Assessment of influencing factors on prescription practices of physicians in bangladesh. International Research Journal of Pharmacy. 2013;4(8):112-6.
39. Ichikawa I, Clayton EW. Doping Doctors: The Influence of the Marketing Departments of Pharmaceutical Companies on Physician and Researcher Behavior in Japan. Account Res. 2016;23(4):245-53.
40. Iizuka T. Experts' agency problems: evidence from the prescription drug market in Japan. Rand J Econ. 2007;38(3):844-62.
41. Indhumathi D, Krishnaraj. The inference of the characteristics of phamaceutical drug advertising upon the level of physician's acceptance. International Journal of Pharmaceutical Sciences Review and Research. 2015;32(2):220-6.
42. Joyce GF, Carrera MP, Goldman DP, Sood N. Physician prescribing behavior and its impact on patient-level outcomes. Am J Manag Care. 2011;17(12):e462-71.

43. Kamal S, Holmberg C, Russell J, Bochenek T, Tobiasz-Adamczyk B, Fischer C, et al. Perceptions and Attitudes of Egyptian Health Professionals and Policy-Makers towards Pharmaceutical Sales Representatives and Other Promotional Activities. PLoS ONE. 2015;10(10):e0140457.
44. Kannan S, Gowri S, Tyagi V, Kohli S, Jain R, Kapil P, et al. Direct-to-physician and direct-to-consumer advertising: Time to have stringent regulations. Int. 2015;27(2):77
45. Khajuria A, Khajuria, V. Impact of Pharmaceutical Marketing Communication Strategies on Prescription Practices of Physicians. RJPBCS. 2013;4(3):882-9.
46. Kinoshita K, Tsugawa Y, Barnett PB, Tokuda Y. Challenging cases of professionalism in Japan: improvement in understanding of professional behaviors among Japanese residents between 2005 and 2013. BMC Med Educ. 2015;15:42.
47. Klemenc-Ketis Z, Kersnik J. The assessment of pharmaceutical sales representatives by family physicians--does it affect the prescribing index? Family practice. 2013;30(3):320-4.
48. Knudsen HK, Roman PM. The transition to medication adoption in publicly funded substance use disorder treatment programs: organizational structure, culture, and resources. Journal of studies on alcohol and drugs. 2014;75(3):476-85.
49. Lee D, Begley CE. Physician report of industry gifts and quality of care. Health Care Manage Rev. 2016;41(3):275-83.
50. Lenzen LM, Weidringer JW, Ollenschlager G. [Conflict of interest in continuing medical education - Studies on certified CME courses]. Z. 2016;110-111:60-8.
51. Lenzer J. California doctors under investigation for prescribing practices. Bmj. 2015;350:h74.
52. Lurk JT, DeJong DJ, Woods TM, Knell ME, Carroll CA. Effects of changes in patient cost sharing and drug sample policies on prescription drug costs and utilization in a safety-net-provider setting. American journal of health-system pharmacy : AJHP : official journal of the American Society of Health-System Pharmacists. 2004;61(3):267-72.
53. Lussier MT, Vanier MC, Authier M, Diallo FB, Gagnon J. Drug sample management in University of Montreal family medicine teaching units. Canadian Family Physician. 2015;61(9):e417-e24.
54. Mackowiak JI, Gagnon JP. Effects of promotion on pharmaceutical demand. Social science & medicine. 1985;20(11):1191-7.
55. Maeda K, Katashima R, Ishizawa K, Yanagawa H. Japanese Physicians' Views on Drug Post-Marketing Surveillance. J Clin Med Res. 2015;7(12):956-60.
56. Mansfield PR. Banning all drug promotion is the best option pending major reforms. J Bioeth Inq. 2005;2(2):75-81.
57. Mansfield PR. Do advertisements in clinical software influence prescribing? Med J Aust. 2008;188(1):13-4.
58. McMahon GT. Advancing Continuing Medical Education. Jama. 2015;314(6):561-2.
59. McGavock H, Webb CH, Johnston GD, Milligan E. Market penetration of new drugs in one United Kingdom region: implications for general practitioners and administrators. Bmj. 1993;307(6912):1118-20.
60. Miao-Sheng C, Yu-Ti S. Pricing of prescription drugs and its impact on physicians' choice behavior. Health Care Manag Sci. 2008;11(3):288-95.
61. Narendran R, Narendranathan M. Influence of pharmaceutical marketing on prescription practices of physicians. Journal of the Indian Medical Association. 2013;111(1):47-50.
62. Newton-Syms FAO, Dawson PH, Cooke J, Feely M, Booth TG, Jerwood D, et al. The influence of an academic representative on prescribing by general practitioners. British Journal of Clinical Pharmacology. 1992;33(1):69-73.
63. O'Dowd A. Doctors face compulsory system to declare gifts and hospitality from drug companies. Bmj. 2015;351:h4617.
64. Peay MY, Peay ER. Differences among practitioners in patterns of preference for information sources in the adoption of new drugs. Social science & medicine. 1984;18(12):1019-25.

65. Prosser H, Walley T. New drug uptake: qualitative comparison of high and low prescribing GPs' attitudes and approach. Family practice. 2003;20(5):583-91.
66. Prosser H, Walley T. Understanding why GPs see pharmaceutical reprensentatives: A qualitative interview study. British Journal of General Practice. 2003;53(489):305-11.

67. Rahman MH, Agarwal S, Tuddenham S, Peto H, Iqbal M, Bhuiya A, et al. What do they do? Interactions between village doctors and medical representatives in Chakaria, Bangladesh. Int Health. 2015;7(4):266-71.
68. Ridley DB. Payments, promotion, and the purple pill. Health economics. 2015;24(1):86-103.
69. Riese F, Guloksuz S, Roventa C, Fair JD, Haravuori H, Rolko T, et al. Pharmaceutical industry interactions of psychiatric trainees from 20 European countries. Eur Psychiatry. 2015;30(2):284-90.
70. Sarpatwari A, Choudhry NK, Avorn J, Kesselheim AS. Paying Physicians to Prescribe Generic Drugs and Follow-On Biologics in the United States. PLoS Medicine. 2015;12 (3) (no pagination)(e1001802).
71. Sharma M, Johnson ML, Vadhariya A, Marcum ZA, Holmes HM. The association of prescriber characteristics with prescriptions for proton pump inhibitors in the medicare part D beneficiaries. Value in Health. 2016;19 (3):A317.
72. Skipper N, Vejlin R. Determinants of generic vs. brand drug choice: evidence from population-wide Danish data. Soc Sci Med. 2015;130:204-15.
73. Spurling G, Mansfield P. General practitioners and pharmaceutical sales representatives: quality improvement research. Qual Saf Health Care. 2007;16(4):266-70.
74. Spurling GK, Mansfield PR, Montgomery BD, Lexchin J, Doust J, Othman N, et al. Information from pharmaceutical companies and the quality, quantity, and cost of physicians' prescribing: a systematic review. PLoS Med. 2010;7(10):e1000352.
75. Stark TJ, Brownell AK, Brager NP, Berg A, Balderston R, Lockyer JM. Exploring Perceptions of Early-Career Psychiatrists About Their Relationships With the Pharmaceutical Industry. Acad Psychiatry. 2016;40(2):249-54.
76. Steinman MA, Harper GM, Chren MM, Landefeld CS, Bero LA. Characteristics and impact of drug detailing for gabapentin. PLoS Med. 2007;4(4):e134.
77. Stelfox HT, Chua G, O'Rourke K, Detsky AS. Conflict of interest in the debate over calcium-channel antagonists. N Engl J Med. 1998;338(2):101-6.
78. Stoddard JJ, Reed M, Hadley J. Financial incentives and physicians' perceptions of conflict of interest and ability to arrange medically necessary services. J Ambulatory Care Manage. 2003;26(1):39-50.
79. Tahmasebi N, Kebriaeezadeh A. Evaluation of factors affecting prescribing behaviors, in iran pharmaceutical market by econometric methods. Iran. 2015;14(2):651-6.
80. Turner P. Profitability and improved patient care - The physician's viewpoint. British Journal of Clinical Pharmacology. 1986;22(SUPPL. 1):41S-3S.
81. Tahmasebi N, Kebriaeezadeh A. Evaluation of factors affecting prescribing behaviors, in iran pharmaceutical market by econometric methods. Iranian journal of pharmaceutical research : IJPR. 2015;14(2):651-6.
82. Taylor RJ, Bond CM. Change in the established prescribing habits of general practitioners: an analysis of initial prescriptions in general practice. British Journal of General Practice. 1991;41(347):244-8.
83. Tengilimoglu D, Kisa A, Ekiyor A. The pharmaceutical sales rep/physician relationship in Turkey: ethical issues in an international context. Health Mark Q. 2005;22(1):21-39.
84. Tobin L, de Almedia Neto AC, Wutzke S, Patterson C, Mackson J, Weekes L, et al. Influences on the prescribing of new drugs. Aust Fam Physician. 2008;37(1-2):78-80, 3.
85. Wilson CWAB, J.A.; Mapes, R.E.A.; Korte, S. pattern of prescribing in general practice British

Medical Journal 1963:604-7.
