# Supplementary material for: Association between physicians’ interaction with pharmaceutical companies and their clinical practices: A systematic review and meta-analysis
Source: PLoS One. 2017 Apr 13;12(4):e0175493. doi: 10.1371/journal.pone.0175493 (PMC5391068; doi:10.1371/journal.pone.0175493)
Supplement: S3 Table — (PDF) [file pone.0175493.s006.pdf]

S3 Table: Challenges and their resolution with regards to including statistical data in the meta-analysis

**Study Name**	**Exposure**	**Outcome**	**Relevant statistical results as reported in the paper**	**Challenge and resolution**
Bowman, 1988 [1]	Attendance of continuing medical education courses	Prescription pattern of sponsored drug for each course	**Course I**: Nifedipine was the drug of the sponsoring company.<br>**Course II**: Metoprolol was the drug of the sponsoring company.<br>**Course III**: Diltiazem was the sponsoring company's drug.<br>Number of new prescriptions for Diltiazem increased statistically from 31.4% (n=257.5) pre-course to 50.1% (n=355.5) post course (p<0.05%)<br>Number of respondents prescribing Diltiazem most frequently to new patients statistically increased from 22.3% to 33.9% pre-post course(p<0.05)	Challenge 1: paper reported results for the same type of exposure (CME) but to different drug (three separate courses); same group of participants.<br><br>Resolution 1: we excluded the results for courses I and II, given data were not matched. We included in the meta-analysis only the results for course III<br><br>Challenge 2: results reported as the number of new prescriptions for Diltiazem and as prescribing Diltiazem most frequently to new patients<br><br>Resolution 2: we considered results for prescribing Diltiazem most frequently to new patients as it provides a more direct measure of the outcome of interest. Furthermore, sensitivity analyses using prescribing Diltiazem most frequently to new patients as the outcome demonstrated no important changes in results (data not shown).
Chren, 1994 [2]	Study assessed three types of exposures:<br>-Detailing<br>-Payment to attend educational symposia<br>-Payment to speak at educational symposia	Addition to formulary	Physicians who had met with pharmaceutical representatives were significantly more likely than other physicians to have requested that drugs manufactured by specific companies be added to the formulary (OR 3.4; 95% CI 1.8-6.6).<br><br>Increased odds of formulary requests were obtained for physicians who had accepted money from those companies to attend educational symposia (OR 7.9; 95% CI 1.1-55.6),	Challenge: paper reported results for three different types of exposures; same group of participants<br><br>Resolution: we treated each exposure as a separate unit of analysis

			Increased odds of formulary requests were obtained for physicians who had accepted money from those companies to speak at educational symposia (OR 3.9; 95% CI 1.2-12.7).	
Figuiras 2000 [3]	Physicians' perception of quality of visiting marketer's information	Quality of drug prescribed: 3 different indicators combined to produce a global indicator	The influence of physicians' perception of quality of visiting marketer's information on the global indicator was significant: co-efficient regression(adjusted for independent variable) :0.178 95% CI(0.037,0.319) p 0.013<br><br>Physician's perception of quality of visiting marketer's information influenced the % of prescribed drugs not included in the formulary for PHC: Adjusted co-efficient regression: 1.021, 95% CI (0.257, 1.784), p 0.009	Challenge: exposure not consistent with the exposure of interest in the meta-analysis; perception of quality of visiting is considered an intermediate outcome<br><br>Resolution: report narratively but not include in the meta-analysis
Orlowski, 2002 [4]	CME	Prescription behavior	Average usage of drug A before the course:<br>81 ± 44 units per month for 22 month before the course<br>-Average usage of drug A after the course:<br>272±117 units (p<O.OO1) for 17 month after the course<br><br>-Average usage of drug B before the second course:<br>34 ± 30 units per month<br>-Average usage of drug B after the course: 87 ± 24 units	Challenge 1: paper reported results for the same exposure but to different drugs (2 CME courses); different group of participants<br><br>Resolution 1: given the data related to two different groups, we considered data for the different drugs separately.<br><br>Challenge 2: reported data for a continuous variable as means and SD, which did not allow including in a meta-analysis of binary variables.<br><br>Resolution 2: although this study is eligible for inclusion in a meta-analysis for continuous data, it is the only study with appropriate continuous data for meta-analysis, thus, we opted to report narratively
Mujrer 2005 [5]	Detailing	Adherence to guidelines for qualitatively good prescribing	More frequent visits from pharmaceutical industry representatives were found to have a significant negative correlation with adherence to guidelines for qualitatively good prescribing β: –0.23 95% CI: (–0.32; –0.15) at p<0.05	Challenge: reported data for a continuous variable as parameter estimate, which did not allow including in a meta-analysis of binary variables.<br><br>Resolution: report narratively as statistical data was presented in different format from Orlowski et al to be

				included in a meta-analysis for continuous data
Miller, 2008 [6]	Presence of sample closet	Generic prescriptions	Two factors were associated with generic prescribing in logistic regression: the absence of drug samples (OR 4.54, 95% CI 1.37–15.0) and the prescriber being an attending physician (OR 5.26, 95% CI 2.24 –12.4).	Challenge 1: odds ration not available for Medicaid patients, only for uninsured patients<br><br>Resolution 1: we considered the odds ratio for uninsured patients<br><br>Challenge 2: unadjusted and adjusted odds ratios<br><br>Resolution 2: we considered the adjusted odds ratio. Furthermore, sensitivity analyses demonstrated no important changes in results (data not shown.
Søndergaard, 2009 [7]	Detailing	Company-specific drug preferences measured as the proportion of dispensing of the promoted drug among all dispensings	The first visit had a statistically significant effect on the GPs' drug preference in favor of the marketed drug [odds ratio (OR), 2.39; 95% confidence interval (CI), 1.72–3.32].<br><br>The effect on drug preference increased further after the second visit (OR, 1.51; 95% CI, 1.19–1.93), while there was no significant change after the third visit (OR, 1.06; 95% CI, 0.94–1.20).	Challenge: post exposure results reported for three different visits.<br><br>Resolution: we considered only the data for the first visit given analysis for subsequent visits is likely to be confounded by the effect of the previous visits, as discussed by the authors: "the effect of promotional visits could in part be caused by representatives selecting practices with a higher probability of adopting the promoted drug. Although we have controlled for the time until first visit, a selection effect cannot be excluded."
Anderson, 2009 [8]	Eating industry-funded food<br><br>Giving drug samples to patients	Reliance on sales representatives when making prescribing decisions	-The frequency of eating industry-funded food (β= .16, 95% CI: .02, .31) and giving drug samples to patients (β= .24, 95% CI: .13, .36) are independently associated with greater reliance on pharmaceutical representatives for drug information when prescribing new medications.<br><br>When the perceived value of pharmaceutical representative is held constant, frequency of drug sample remained independently associated with greater reliance on pharmaceutical representatives for drug information	Challenge 1: 'giving drug samples to patients' was treated as an exposure by the authors.<br><br>Resolution 2: we did not include consider this analysis.<br><br>Challenge 2: the outcome 'Reliance on sales representatives' is a surrogate for our outcome of interest (behavior) in the meta-analysis. Also the authors adjusted for perceived value

			when prescribing new medication (β= .16, 95% CI: .06, .26).	of pharmaceutical representative when studying the association between eating industry-provided food and reliance on pharmaceutical representatives for drug information.<br><br>Resolution 2: we reported the findings narratively but not include in the meta-analysis.
Pinckney 2010 [9]	Drug samples	Prescription preference in response to two clinical vignettes	Clinicians with samples available were less likely to prescribe thiazide diuretics [OR=0.2 (95% CI 0.06–0.68)]. To test the robustness of this conclusion, authors conducted a full regression which showed that clinicians with samples were still less likely to select a thiazide diuretic [OR=0.15 (95% CI 0.04–0.56)].<br><br>Primary care prescribers who dispensed samples at least weekly were less likely to select a thiazide in the vignette than those that dispensed samples less frequently or not at all [OR=0.4 (95% CI 0.18–0.85)].	Challenge 1: paper reported both unadjusted and adjusted odds ratios<br><br>Resolution 1: we included the odds ratio for the presence of drug samples after conducting full regression. Furthermore, sensitivity analyses demonstrated no important changes in results (data not shown).<br><br>Challenge 2: full regression was only possible for the hypertension vignette<br><br>Resolution 2: we included the adjusted odds ratio for the hypertension vignette<br>Challenge 3: paper reports data for behavior (outcome of interest in our review) treated as an exposure.<br><br>Resolution 3: we did not consider the data about giving drug samples to patients as this variable was treated as an exposure by the authors.
Pedan, 2011 [10]	Detailing<br><br>Sampling<br><br>Free meals	Prescription pattern	Detailing produced a highly significant positive effect on new prescriptions for Lipitor 0.098±0.041 $p<0.05$ and Crestor 0.132±0.054 $p<0.05$, but results were not significant for Vytorin.<br><br>Sample dispensing had a significant positive effect for Crestor 0.106 ± 0.027 $p<0.01$ and Vytorin 0.101±0.04 $p<0.05$. Results were not significant for Lipitor. Free meals had a significant positive impact on all three strain brands: Lipitor	Challenge: reported data for a continuous variable as parameter estimate, which did not allow including in a meta-analysis of binary variables.<br><br>Resolution: report narratively as statistical data was presented in different format from Orlowski et al to be

			0.097±0.04 p<0.05, Crestor 0.066±0.032 p<0.05 and Vytorin 0.163±0.039 p<0.01.	included in a meta-analysis for continuous data
Lieb 2014 [11]	CME<br><br>Acceptance of gifts	Individual prescribing of physicians over a year for all on-patent branded, off-patent branded, and generic drugs prescription	Compared to doctors who frequently, occasionally or rarely took part in sponsored CME events, doctors who mentioned they never took part in such events had a lower number of on patent-branded drug prescriptions per patient (mean ± SD; 1.05±0.35 vs. 1.27±0.55; p= 0.005, a higher proportion of generics (83.28±7.77% vs. 76.34±13.58%; p<0.0005)<br><br>Physicians who always or frequently accepted office stationery prescribed higher daily dose totals per patient (mean ± SD; 491.97±158.95 vs. 420.53±140.57; p = 0.003) and more generics (mean ±SD; 385.52±147.52 vs. 319.43±133.69; p =0.004) in comparison to physicians who only occasionally, rarely or never accepted stationery. (n=97)	Challenge: the categorization of answer options was not conducive to interpretation. For e.g. when comparing influence of CME, authors treated physicians who 'frequently' 'occasionally', or 'rarely' took part in CME events as exposure group and physicians who 'never' took part as control. In contrast, when comparing influence of gifts, authors treated physicians who 'always' or 'frequently' accepted gifts as exposure group and physicians who 'occasionally', 'rarely' or 'never' accepted as control group<br><br>Resolution: report narratively and noted the challenge in interpretation
Dejong 2016 [12]	Industry meal	Prescription behavior	Physicians who received a single meal promoting the drug of interest had higher rates of prescribing of rosuvastatin over other statins (odds ratio (OR), 1.18; 95% CI, 1.17-1.18); nebivolol over other β-blockers (OR, 1.70; 95% CI, 1.69-1.72); olmesartan over other ACE inhibitors and ARBs (OR, 1.52; 95% CI, 1.51-1.53); and desvenlafaxine over other SSRIs and SNRIs (OR, 2.18; 95% CI, 2.13-2.23).	Challenge 1: paper reported results for the same exposure but to 4 different drugs; same group of participants<br><br>Resolution 1: Given the data related to the same group, we calculated the average OR for the different drugs and took the value closest to it. (i.e. OR, 1.70; 95% CI, 1.69-1.72)
Yeh 2016 [13]	Industry payments for different types of gifts (e.g. meal; grants/educational gifts ; educational training)	Behavior: Rate of prescribing brand-name statins as compared with generic statins for lowering cholesterol	Among physicians with industry payments reported in the Massachusetts database, every $1000 in total payments received was associated with a 0.1% increase in the rate of brand-name statin drug prescribing (95% CI, 0.06%-0.13%; P < .001).<br>Payments for educational training were associated with an average 4.8% increase in brand-name prescribing compared with no receipt of educational training (95% CI, 1.55-7.95;P = .004), but the other payment types were not.	Challenge: reported data for a continuous variable as parameter estimate, which did not allow including in a meta-analysis of binary variables.<br><br>Resolution: report narratively as statistical data was presented in different format from Orlowski et al to be included in a meta-analysis for continuous data

References

1. Bowman MA, Pearle DL: **Changes in drug prescribing patterns related to commercial company funding of continuing medical education**. *J Contin Educ Health Prof* 1988, **8**(1):13-20.
2. Chren MM, Landefeld CS: **Physicians' behavior and their interactions with drug companies. A controlled study of physicians who requested additions to a hospital drug formulary**. *Jama* 1994, **271**(9):684-689.
3. Figueiras A, Caamano F, Gestal-Otero JJ: **Influence of physician's education, drug information and medical-care settings on the quality of drugs prescribed**. *Eur J Clin Pharmacol* 2000, **56**(9-10):747-753.
4. Orlowski JP, Wateska L: **The effects of pharmaceutical firm enticements on physician prescribing patterns. There's no such thing as a free lunch**. *Chest* 1992, **102**(1):270-273.
5. Muijrers PE, Grol RP, Sijbrandij J, Janknegt R, Knottnerus JA: **Differences in prescribing between GPs: impact of the cooperation with pharmacists and impact of visits from pharmaceutical industry representatives**. *Fam Pract* 2005, **22**(6):624-630.
6. Miller DP, Mansfield RJ, Woods JB, Wofford JL, Moran WP: **The impact of drug samples on prescribing to the uninsured**. *South Med J* 2008, **101**(9):888-893.
7. Sondergaard J, Vach K, Kragstrup J, Andersen M: **Impact of pharmaceutical representative visits on GPs' drug preferences**. *Fam Pract* 2009, **26**(3):204-209.
8. Anderson BL, Silverman GK, Loewenstein GF, Zinberg S, Schulkin J: **Factors associated with physicians' reliance on pharmaceutical sales representatives**. *Acad Med* 2009, **84**(8):994-1002.
9. Pinckney RG, Helminski AS, Kennedy AG, Maclean CD, Hurowitz L, Cote E: **The effect of medication samples on self-reported prescribing practices: a statewide, cross-sectional survey**. *J Gen Intern Med* 2011, **26**(1):40-44.
10. Pedan A, Wu H: **Asymmetric responsiveness of physician prescription behavior to drug promotion of competitive brands within an established therapeutic drug class**. *Health Mark Q* 2011, **28**(2):133-154.
11. Lieb K, Scheurich A: **Contact between doctors and the pharmaceutical industry, their perceptions, and the effects on prescribing habits**. *PLoS One* 2014, **9**(10):e110130.
12. DeJong C, Aguilar T, Tseng CW, Lin GA, Boscardin WJ, Dudley RA: **Pharmaceutical Industry-Sponsored Meals and Physician Prescribing Patterns for Medicare Beneficiaries**. *JAMA Intern Med* 2016, **176**(8):1114-1110.
13. Yeh JS, Franklin JM, Avorn J, Landon J, Kesselheim AS: **Association of Industry Payments to Physicians With the Prescribing of Brand-name Statins in Massachusetts**. *JAMA Intern Med* 2016, **176**(6):763-768.
