# Supplementary figures and images for: Association between physicians’ interaction with pharmaceutical companies and their clinical practices: A systematic review and meta-analysis
Source: PLoS One. 2017 Apr 13;12(4):e0175493. doi: 10.1371/journal.pone.0175493 (PMC5391068; doi:10.1371/journal.pone.0175493)

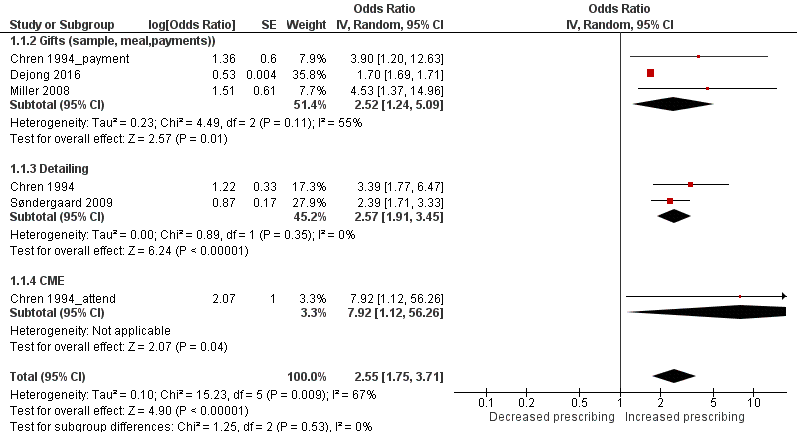

Supplement: S1 Fig — (TIF) [file pone.0175493.s007.tif]

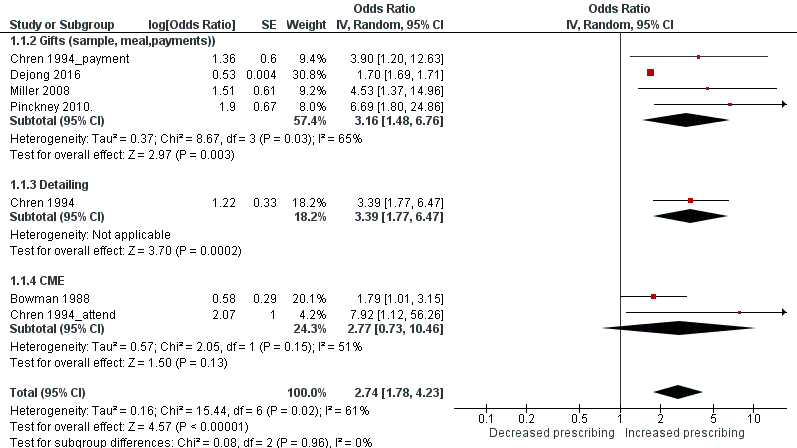

Supplement: S2 Fig — (TIF) [file pone.0175493.s008.tif]

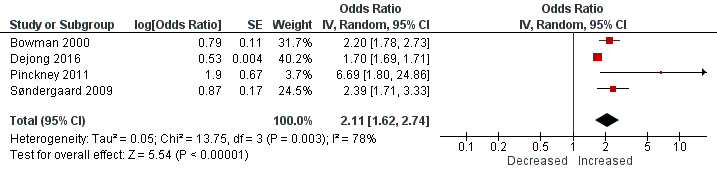

Supplement: S3 Fig — (TIF) [file pone.0175493.s009.tif]
